# Constructing CoP/Ni_2_P Heterostructure Confined Ru Sub‐Nanoclusters for Enhanced Water Splitting in Wide pH Conditions

**DOI:** 10.1002/advs.202401398

**Published:** 2024-07-11

**Authors:** Huimin Zhang, Wenhao Liu, Zhenhao Li, Liang Qiao, Kebin Chi, Xiaoyan Guo, Dong Cao, Daojian Cheng

**Affiliations:** ^1^ State Key Laboratory of Organic‐Inorganic Composites College of Chemical Engineering Beijing University of Chemical Technology Beijing 100029 P. R. China; ^2^ PetroChina Petrochemical Research Institute Beijing 102206 P. R. China

**Keywords:** alkaline water electrolyzer, heterojunction, interface confinement, Ru‐CoP/Ni_2_P, water splitting

## Abstract

Developing efficient electrocatalysts for water splitting is of great significance for realizing sustainable energy conversion. In this work, Ru sub‐nanoclusters anchored on cobalt‐nickel bimetallic phosphides (Ru‐CoP/Ni_2_P) are constructed by an interfacial confinement strategy. Remarkably, Ru‐CoP/Ni_2_P with low noble metal loading (33.1 µg cm^−2^) shows superior activity for hydrogen evolution reaction (HER) in all pH values, whose turnover frequency (TOF) is 8.7, 15.3, and 124.7 times higher than that of Pt/C in acidic, alkaline, and neutral conditions, respectively. Meanwhile, it only requires the overpotential of 171 mV@10 mA cm^−2^ for oxygen evolution reaction (OER) and corresponding TOF is 20.3 times higher than that of RuO_2_. More importantly, the Ru‐CoP/Ni_2_P||Ru‐CoP/Ni_2_P displays superior mass activity of 4017 mA mg_noble metal_^−1^ at 2.0 V in flowing alkaline water electrolyzer, which is 105.1 times higher than that of Pt/C||IrO_2_. In situ Raman spectroscopy demonstrates that the Ru sites in Ru‐CoP/Ni_2_P play a key role for water splitting and follow the adsorption evolution mechanism toward OER. Further mechanism studies disclose the confined Ru atom contributes to the desorption of H_2_ during HER and the formation of O‐O bond during OER, leading to fast reaction kinetics. This study emphasizes the importance of interface confinement for enhancing electrocatalytic activity.

## Graphical abstract

The Ru sub‐nanoclusters confined on the CoP/Ni_2_P herterogenous structure show superior electrocatalytic performance for pH‐universal HER and alkaline OER. In situ Raman and DFT calculations reveal that the confinement effect endows superior intrinsic activity to Ru‐CoP/Ni_2_P, and the active Ru sites contributes to the desorption of H_2_ for HER and promoting the transformation of O* to OOH* for OER.

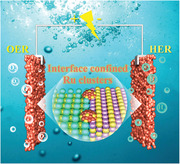

## 1 Introduction

Hydrogen (H_2_) is considered as an attractive candidate for renewable energy owing to its versatility, cleanliness, high energy density, and environmental friendliness.^[^ ^1^, ^2^ ^]^ Among the diverse approaches to hydrogen production, electrochemical water splitting has received considerable attention as a promising method with zero carbon production.^[^ ^3^, ^4^, ^5^ ^]^ The overall water splitting (OWS) involves two half‐reactions, including hydrogen evolution reaction (HER) on the cathode and oxygen evolution reaction (OER) on the anode, in which Pt/C and RuO_2_ are considered as the state‐of‐the‐art electrocatalysts, respectively.^[^ ^6^, ^7^ ^]^ However, the scarcity and sluggish kinetics of noble metals limit the large‐scale development.^[^ ^8^, ^9^, ^10^ ^]^ Moreover, the development of electrocatalysts with both superior HER and OER performance is of great practical application significance.^[^ ^11^ ^]^ Thus, it is imperative to develop high‐performance and cost‐efficient low‐noble metal electrocatalysts for OWS.

Ruthenium (Ru) with only 1/4 price of Pt has attracted a wide interest, showing economic merits and favoring water dissociation.^[^ ^12^, ^13^, ^14^ ^]^ However, Ru‐based catalysts typically exhibit unsatisfied stability and inefficient reaction process due to the high dissolution rate and strong adsorption of OH.^[^ ^15^, ^16^ ^]^ The application of metal‐support interaction is one of the effective strategies to reduce the content of noble metal and improve the intrinsic activity of active sites by integrating noble metals into support.^[^ ^17^, ^18^, ^19^, ^20^ ^]^ Supports play a key role in anchoring the active metal and stabilizing it from agglomeration, which is conducive to improve the dispersion and stability of catalysts.^[^ ^21^, ^22^, ^23^ ^]^ Meanwhile, a superior support can affect and even regulate the materials properties due to the metal‐substrate electronic interactions.^[^ ^24^ ^]^ Carbon and relevant species, metal oxides, alloys, and so on, have been studied as supports.^[^ ^25^, ^26^, ^27^, ^28^ ^]^ For instance, Liu and co‐workers designed the Ru nanoclusters anchored on N‐doped ultrathin carbon nanosheets. The interaction between Ru and N‐doped carbon leads to the enhanced electrocatalytic performance for HER.^[^ ^29^ ^]^ More importantly, the heterogeneous interfaces exhibit unique properties over their single components by virtue of interfacial coupling effect.^[^ ^30^ ^]^ Constructing well‐defined heterojunctions as supports is proposed to be an effective way to enhance the reactivity, which can not only improve the interaction on active sites by facilitating electron transfer between metal and support, but also modulate the adsorption/desorption energy of reaction intermediates.^[^ ^31^, ^32^ ^]^ Furthermore, the interface confinement effect shows an essential effect in regulating the reactivity sites and enhancing the intrinsic activity.^[^ ^33^, ^34^, ^35^ ^]^ However, there are a few relevant researches about the confinement effect between heterointerfaces and Ru clusters. Thus, it is imperative to design the confined Ru clusters on the support of heterostructure and explore the metal‐support interaction.

Herein, Ru clusters are confined on the heterostructure of cobalt‐nickel bimetallic phosphide (Ru‐CoP/Ni_2_P) through a sequential phase conversion process. The morphology of resulting Ru‐CoP/Ni_2_P is modulated as the tip‐twisted nanoneedles, facilitating the electron transfer and ensuring large specific active area. Importantly, the as‐prepared Ru‐CoP/Ni_2_P displays enhanced electrochemical performance for pH‐universal HER and alkaline OER. The confinement effect endows superior intrinsic activity to Ru‐CoP/Ni_2_P, whose turnover frequency (TOF) values of HER are 15.3, 124.7, and 8.7 times higher than that of Pt/C under basic, neutral, and acidic conditions, respectively. Meanwhile, the TOF value of Ru‐CoP/Ni_2_P for OER is 20.3 times higher than that of RuO_2_, and it also displays an excellent performance in alkaline water electrolyzer (AWE). In situ Raman and density functional theory (DFT) reveal that Ru atoms act as the main active sites during HER and OER, which contributes to the desorption of H_2_ during HER. Meanwhile, Ru sites accelerate the dissociation of water and promote the conversion from O* to OOH* during OER. This work develops a potential electrocatalyst for practical application and sheds light on the interface confinement effect for boosting catalytic performance.

## 2 Results and Discussion

### 2.1 Structural Characterizations

**Figure 1:**
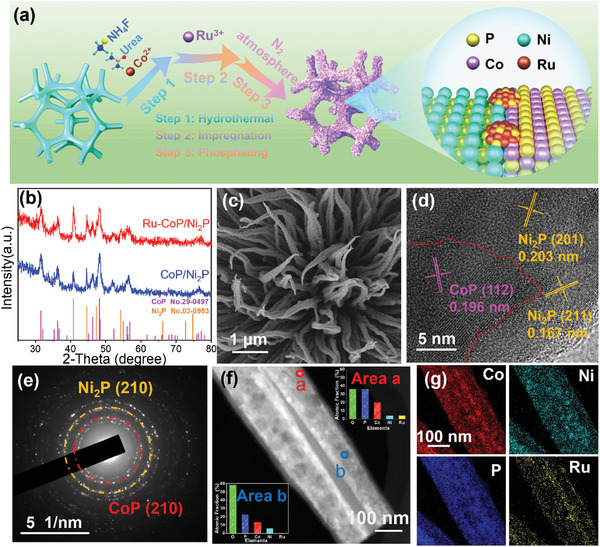
a) Schematic diagram of the synthesis process of Ru‐CoP/Ni_2_P. b) XRD patterns of Ru‐CoP/Ni_2_P and CoP/Ni_2_P. c) SEM image of Ru‐CoP/Ni_2_P, d) HRTEM image of Ru‐CoP/Ni_2_P. e) The SAED pattern of Ru‐CoP/Ni_2_P. f) HAADF‐STEM image of Ru‐CoP/Ni_2_P. g) EDS element mapping images of Ru‐CoP/Ni_2_P nanorods.

As shown in **Figure** **1**a, Ru‐CoP/Ni_2_P of cobalt‐nickel bimetallic phosphides doped with noble metal ruthenium were prepared by a simple hydrothermal post‐impregnation method coupled with low‐temperature phosphating. First, Cobalt(II) carbonate hydroxide (Co(CO_3_)_0.5_(OH)·0.11H_2_O) was supported on the nickel foam through hydrothermal process, which was proved by X‐ray diffraction (XRD) (Figure S1, Supporting Information). Then Ru^3+^ was introduced into the sample through impregnation method. Finally, Ru‐CoP/Ni_2_P was successfully prepared after low‐temperature phosphating process. For comparison, CoP/Ni_2_P, CoP, and Ni_2_P were synthesized under same conditions by adjusting some steps (see details in Supporting Information). XRD was employed to analyze the phase composition of the samples. For CoP, a single set of diffraction peaks at 2θ = 31.6°, 36.3°, 46.2°, and 48.1° correspond to the (011), (111), (112), and (211) crystal planes of CoP (JCPDS No. 29–0497) (Figure S2, Supporting Information). Figure S3 (Supporting Information) shows the peaks located at 40.8°, 44.6°, 47.3°, and 54.2°, which matches well with the (111), (201), (210), and (300) facets of Ni_2_P (JCPDS No. 03–0953), respectively. The XRD pattern of as‐prepared Ru‐CoP/Ni_2_P possesses the same diffraction peaks as CoP/Ni_2_P (Figure 1b), exhibiting the phase of Ni_2_P and CoP. Notably, the absence of peaks associated with Ru species in Ru‐CoP/Ni_2_P could be attributed to the presence of Ru in the form of clusters. Furthermore, scanning electron microscopy (SEM) was applied to observe the morphology of the samples. As depicted in Figures S4 and S5 (Supporting Information), Ni_2_P shows a disordered and stacked structure and CoP exhibits the morphology of irregular nanosheet. As shown in Figure 1c and Figure S6 (Supporting Information), Ru‐CoP/Ni_2_P exhibits a well‐defined nanoneedle structure, which provides numerous catalytically active sites over CoP and Ni_2_P. Compared with CoP/Ni_2_P without the impregnation process (Figure S7, Supporting Information), Ru‐CoP/Ni_2_P possesses the unique tip‐twisted feature, which is conductive to enhance the intrinsic activity of the catalyst. Transmission electron microscopy (TEM) images indicate that the nanoneedle structure is ranging from 50–100 nm in diameter (Figure S8, Supporting Information). Further high‐resolution TEM (HRTEM) analysis (Figure 1d) reveals the existence of distinct interfaces. The measured lattice spacings of 0.196, 0.167, and 0.203 nm correspond to the CoP (112), Ni_2_P (211), and (201) crystal planes, respectively, confirming the presence of hetero‐interface structure in Ru‐CoP/Ni_2_P. Additionally, HRTEM image (Figure S9, Supporting Information) reveals the presence of high contrast sub‐nanoclusters, which are typically associated with noble metals. It can be seen that the interfaces play a key role in restricting the aggregation of Ru. The selected area electron diffraction (SAED, Figure 1e) image shows the concentric ring patterns, indicating its polycrystalline structure. Meanwhile, the (210) crystal planes of CoP and Ni_2_P further confirm the heterostructure of Ru‐CoP/Ni_2_P. The mass fraction was characterized by inductively coupled plasma spectrometry, demonstrating the low content of 33.1 µg cm^−2^ of Ru (Table S1, Supporting Information). High‐angle annular dark‐field scanning TEM (HAADF‐STEM) was further performed. As shown in Figure 1f, the bright point “a” reveals the existence of a small amount of Ru, while the normal area “b” shows no significant response to Ru signal. Combining the above analyses, Ru exists in the form of clusters in Ru‐CoP/Ni_2_P. Meanwhile, higher‐magnification HAADF‐STEM image of Ru‐CoP/Ni_2_P is shown in Figure S10 (Supporting Information), where the presence of Ru‐containing bright spots can be seen. Furthermore, energy dispersive X‐ray spectroscopy (EDS) elemental mapping images (Figure 1g) suggest the uniform distribution of Co, Ni, P, and Ru elements throughout Ru‐CoP/Ni_2_P.

**Figure 2:**
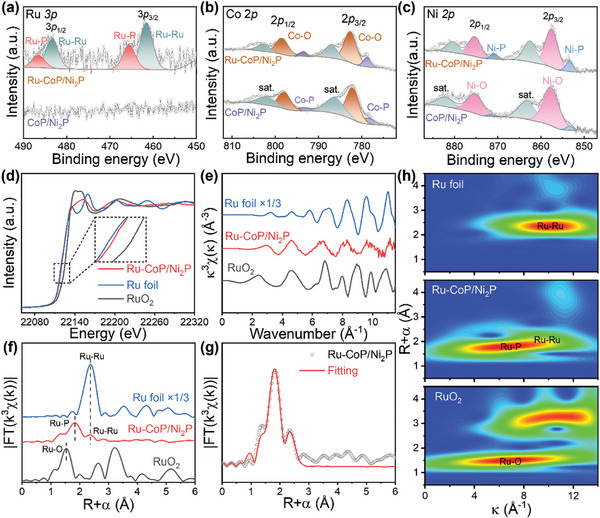
High‐resolution XPS spectra of a) Ru *3p*, b) Co *2p*, and c) Ni *2p* for Ru‐CoP/Ni_2_P and CoP/Ni_2_P. d) Normalized XANES spectra and e) EXAFS spectra at Ru K‐edge of Ru‐CoP/Ni_2_P, Ru foil, and RuO_2_. f) FT‐EXAFS results for Ru‐CoP/Ni_2_P, Ru foil, and RuO_2_. g) *k* ^3^‐weighted FT‐EXAFS fitting curves of Ru‐CoP/Ni_2_P. The data have not been phase‐corrected. h) Wavelet transform EXAFS spectra of Ru‐CoP/Ni_2_P, Ru foil and RuO_2_.

X‐ray photoelectron spectroscopy (XPS) was carried out to further investigate the surface composition and electronic structure of the samples. **Figure** **2**a shows the high‐resolution Ru 3*p* spectra of Ru‐CoP/Ni_2_P and CoP/Ni_2_P. The signal of Ru is only found in Ru‐CoP/Ni_2_P, indicating the successful introduction of Ru to the sample. In detail, the Ru 3*p*_3/2_ peak centered at 461.5 eV and the Ru 3*p*_1/2_ peak centered at 483.3 eV correspond to Ru─Ru bonds, whereas the fitted peaks at 465.5 and 486.5 eV declare the presence of Ru─P bonds.^[^ ^36^ ^]^ The presence of Ru─P and Ru─Ru bonds confirms the formation of Ru clusters. In the Co 2*p* spectrum of Ru‐CoP/Ni_2_P, the peaks located at 778.8 and 793.5 eV correspond to Co─P bonds, while the peaks at 782.6 and 798.4 eV are indexed to the Co─O bonds (Figure 2b).^[^ ^31^ ^]^ The Co─O bonds mainly originate from the surface oxidation of CoP species in Ru‐CoP/Ni_2_P. The peaks located at 787.0 and 802.8 eV correspond to the satellite peaks of Co 2*p*_3/2_ and Co 2*p*_1/2_, respectively.^[^ ^5^ ^]^ For the Ni 2*p* spectrum (Figure 2c), the two peaks with binding energies of 853.4 and 870.8 eV are attributed to the Ni 2*p*_3/2_ and Ni 2*p*_1/2_ orbitals of the Ni‐P bonds, verifying the existence of Ni_2_P. The peaks located at 857.5 and 875.3 eV correspond to the Ni─O bonds, which is also derived from the surface oxidation of the samples.^[^ ^35^ ^]^ Meantime, two corresponding satellite peaks are located at 862.5 and 880.4 eV. Remarkably, the binding energy of Co and Ni spectra in Ru‐CoP/Ni_2_P is 0.1 eV higher than that in CoP/Ni_2_P, indicating the metal‐support interaction and the electron transfer from Co and Ni to Ru. The electron‐rich state of Ru is alleviated to the strong adsorption of OH on Ru atoms, which is conducive to the electrocatalytic process.^[^ ^37^ ^]^ High‐resolution P 2*p* spectra are shown in Figure S11 (Supporting Information). The fitting peaks corresponding to 129.4 and 130.5 eV should be ascribed to the 2*p*_3/2_ and 2*p*_1/2_ of P‐metal, and another peaks located at 134.0 and 134.9 eV indicate the presence of P─O for Ru‐CoP/Ni_2_P.^[^ ^38^ ^]^ The predominate P─O is derived from the oxidation in air.^[^ ^39^ ^]^ Furthermore, the content of P‐metal in Ru‐CoP/Ni_2_P was substantially high compared with those in CoP/Ni_2_P, suggesting the generation of Ru─P during the formation of Ru‐CoP/Ni_2_P.

To further investigate the electronic state and structure information of Ru‐CoP/Ni_2_P, X‐ray absorption spectrum analysis was conducted. The Ru K‐edge X‐ray absorption near edge structure (XANES) spectra of Ru‐CoP/Ni_2_P, Ru foil, and RuO_2_ are shown in Figure 2d. The white line peak displays that the average oxidation state of Ru in Ru‐CoP/Ni_2_P is between Ru foil and RuO_2_ and considerably close to the metallic state, which is consistent with the analyses of XPS results and is more favorable for accelerating the electrochemical kinetics.^[^ ^40^ ^]^ Then, extended X‐ray absorption fine structure (EXAFS) spectroscopy was applied to analyze the coordination environment of Ru atoms. As shown in Figure 2e, the k space reveals the coordination shells of Ru are different from that of Ru foil and RuO_2_. The Fourier transform (FT)‐EXAFS spectrum of Ru‐CoP/Ni_2_P suggests two main peaks at 1.83 and 2.37 Å corresponding to Ru─P and Ru─Ru interactions, which is distinct from the R space of Ru foil and RuO_2_ (Figure 2f). Furthermore, the EXAFS fitting curves and quantitative analysis results are shown in Figure 2g and Table S2 (Supporting Information), which provide the information on detailed parameters of local structure about Ru atoms. The average coordination numbers of Ru─P and Ru─Ru scattering paths in Ru‐CoP/Ni_2_P were evaluated to be 3.4 and 2.8, with mean bond lengths of 2.32 and 2.66 Å, respectively. The structural information was further visualized by wavelet transform (WT) simulation diagrams (Figure 2h). The WT intensity maximum at ≈7.3 Å^−1^ arising from the Ru‐P and Ru‐Ru bonds was well resolved from 1.6 to 2.1 Å for Ru‐CoP/Ni_2_P, which differs from the Ru‐Ru bond of Ru foil at R ≈ 7.2 Å^−1^/k≈ 1.5 Å and the Ru─O bond of RuO_2_ at R ≈ 9.8 Å^−1^/k≈ 2.4 Å. Therefore, the above analyses confirm that the Ru atoms were anchored on the interface of CoP and Ni_2_P with the presence of Ru─P and Ru─Ru bonds.

### 2.2 Electrocatalytic Performance Evaluation

**Figure 3:**
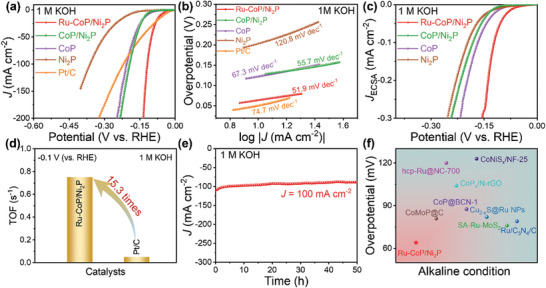
a) HER polarization curves of Ru‐CoP/Ni_2_P, CoP/Ni_2_P, CoP, Ni_2_P, and Pt/C. b) The corresponding Tafel slope plots. c) The current density normalized by ECSA of the samples. d) Comparison of TOF values of Ru‐CoP/Ni_2_P and Pt/C at −0.1 V (vs RHE). e) Time‐dependent current density curve under 100 mA cm^−2^ for 50 h. f) Comparison of the overpotential (η_10_) of Ru‐CoP/Ni_2_P and reported Ru‐based and Co‐based electrocatalysts. The above performance graphs were measured in 1.0 m KOH electrolyte.

The electrochemical performance of as‐prepared samples was evaluated by a standard three‐electrode system with 85% *i*R correction. The linear sweep voltammetry (LSV) curves in **Figure** **3**a show that Ru‐CoP/Ni_2_P achieves an overpotential of 64 mV to reach the current density of 10 mA cm^−2^ in 1.0 m KOH electrolyte, while CoP/Ni_2_P, CoP, and Ni_2_P require overpotentials of 127, 123, and 204 mV, respectively. Additionally, Ru‐CoP/Ni_2_P displays superior catalytic activity at high current density. It only needs an overpotential of only 119 mV at 100 mA cm^−2^, significantly outperforming commercial Pt/C (220 mV) and CoP/Ni_2_P (194 mV). The Ru‐CoP/Ni_2_P catalyst exhibits a smaller Tafel slope of 51.9 mV dec^−1^ compared to Pt/C (74.7 mV dec^−1^), CoP/Ni_2_P (55.7 mV dec^−1^), CoP (67.3 mV dec^−1^), and Ni_2_P (120.8 mV dec^−1^), suggesting that Ru‐CoP/Ni_2_P shows an improved HER kinetics and undergoes a Volmer‐Heyrovsky reaction process in alkaline condition (Figure 3b). These results indicated that the formation of heterojunction and confinement effect contribute to the enhancement of hydrogen evolution performance. The charge‐transfer kinetics were investigated through electrochemical impedance spectroscopy (EIS) measurements. The corresponding equivalent circuit diagram was fitted, showing the solution resistance (R_s_), double layer capacitance (C_dl_) and charge transfer resistance (R_ct_) of the simplified Randles cell. Figure S12 and Table S3 (Supporting Information) show that the charge transfer resistance of Ru‐CoP/Ni_2_P (R_ct_ = 3.6 Ω) is lower than that of Pt/C (R_ct_ = 7.0 Ω), CoP/Ni_2_P (R_ct_ = 65.0 Ω), CoP (R_ct_ = 67.0 Ω), and Ni_2_P (R_ct_ = 252.2 Ω), indicating the faster reaction rate of Ru‐CoP/Ni_2_P. To explore the intrinsic activity of the samples, a series of cyclic voltammetry (CV) curves were recorded in the non‐faradaic potential region. The double‐layer capacitance (C_dl_) values of the samples were fitted as shown in Figure S13 (Supporting Information). Ru‐CoP/Ni_2_P displays the largest C_dl_ value of 45.9 mF cm^−2^, compared to CoP/Ni_2_P (30.5 mF cm^−2^), CoP (16.8 mF cm^−2^), Ni_2_P (3.4 mF cm^−2^), and Pt/C (12.6 mF cm^−2^). Thus, Ru‐CoP/Ni_2_P also exhibits the largest electrochemical active surface area (ECSA) due to the positive correlation between ECSA and C_dl_ (Table S4, Supporting Information). Furthermore, the ECSA‐normalized specific activity (*j*_ECSA_) was calculated and is shown in Figure 3c. Ru‐CoP/Ni_2_P still displays superior electrocatalytic activity than CoP/Ni_2_P, CoP, Ni_2_P, and Pt/C, suggesting that the introduction of Ru leads to the increasing active sites and intrinsic activity. In addition, TOF plots demonstrate that Ru‐CoP/Ni_2_P exhibits a higher intrinsic activity with the TOF value of 0.75 s^−1^ at −0.1 V (vs RHE), which is 15.3 times higher than that of Pt/C (0.049 s^−1^) (Figure 3d; Figure S14, Supporting Information). This further confirms the enhanced intrinsic HER activity of Ru‐CoP/Ni_2_P compared to other samples due to the confinement effect. More importantly, Ru‐CoP/Ni_2_P shows 29.3 times higher mass activity (1433.8 mA mg_Ru_^−1^) than Pt/C (49.0 mA mg_Pt_^−1^) at −0.1 V versus RHE (Figures S15 and S16, Supporting Information). Stability is a vital parameter to evaluate the capacity for practical application of electrocatalysts. The chronoamperometry measurement was carried out and the HER activity of Ru‐CoP/Ni_2_P shows almost no decay at 100 mA cm^−2^ after 50‐h testing, suggesting its superior stability (Figure 3e). In addition, the overpotential of Ru‐CoP/Ni_2_P is also compared with those of reported advanced HER electrocatalysts in alkaline media (Figure 3f; and Table S5, Supporting Information), illustrating that the as‐prepared Ru‐CoP/Ni_2_P exhibited prominent advantages for hydrogen evolution.

**Figure 4:**
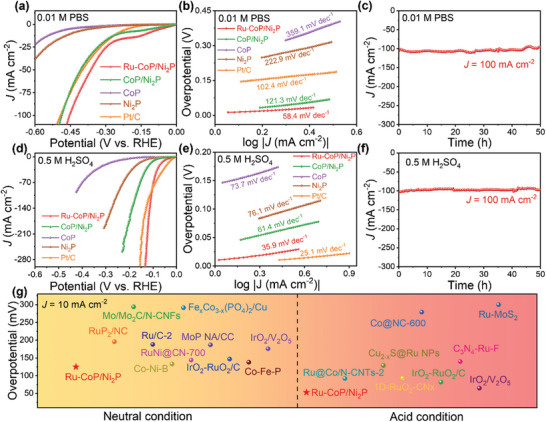
a) HER LSV curves of Ru‐CoP/Ni_2_P, CoP/Ni_2_P, CoP, Ni_2_P, and Pt/C in 0.01 m PBS condition. b) The corresponding Tafel slope plots. c) Time‐dependent current density curve of Ru‐CoP/Ni_2_P at 100 mA cm^−2^ for 50 h in the neutral solution. d) HER LSV curves of Ru‐CoP/Ni_2_P, CoP/Ni_2_P, CoP, Ni_2_P, and Pt/C in 0.5 m H_2_SO_4_ condition. e) The corresponding Tafel slope plots. f) Time‐dependent current density curve of Ru‐CoP/Ni_2_P at 100 mA cm^−2^ for 50 h in the acidic solution. g) Comparison of overpotentials (η_10_) for recently published advanced electrocatalysts in neutral and acid solutions.

What's more, HER performance of the catalysts was tested in 0.01 m phosphate‐buffered saline (PBS) solution. Ru‐CoP/Ni_2_P shows excellent catalytic activity with an overpotential of 125 mV at 10 mA cm^−2^, which is much smaller commercial Pt/C, CoP/Ni_2_P, CoP, and Ni_2_P (**Figure** **4**a). The Tafel slope of Ru‐CoP/Ni_2_P (58.4 mV dec^−1^) is lower than that of other samples (Figure 4b), indicating its faster reaction dynamics. Ru‐CoP/Ni_2_P with the smallest semicircle (R_ct_ = 7.0 Ω) exhibits the fastest electron transfer rate (Figure S17 and Table S6, Supporting Information). Additionally, the plotted C_dl_ for electrocatalysts reveals the ECSA of Ru‐CoP/Ni_2_P is larger than that of CoP/Ni_2_P, CoP, Ni_2_P and Pt/C (Figure S18 and Table S7, Supporting Information). Thus, Ru‐CoP/Ni_2_P exposes more active sites in neutral media. As shown in Figure S19 (Supporting Information), Ru‐CoP/Ni_2_P provides a TOF value of 0.12 s^−1^, which is 124.7 times higher than that of Pt/C, which indicates the exceptional intrinsic activity. Figure S20 (Supporting Information) shows that the mass activity of Ru‐CoP/Ni_2_P (229.5 mA mg^−1^) is 241.2 times higher than that of Pt/C at −0.1 V (vs RHE), suggesting higher utilization efficiency of noble metal. The Ru‐CoP/Ni_2_P displayed negligible attenuation after 50 h long‐term stability test (Figure 4c). Moreover, the hydrogen evolution performance of samples in 0.5 m H_2_SO_4_ was investigated. The polarization curves (Figure 4d) show a low overpotential of 53 mV at 10 mA cm^−2^ of Ru‐CoP/Ni_2_P, outperforming CoP/Ni_2_P, CoP, and Ni_2_P. Besides, Ru‐CoP/Ni_2_P displays superior acidic HER performance over commercial Pt/C at higher current density (> 100 mA cm^−2^). The low Tafel slope of Ru‐CoP/Ni_2_P (35.9 mV dec^−1^) suggests fast catalytic reaction kinetics, explaining its excellent performance under acidic condition (Figure 4e). The Nyquist plots demonstrate that Ru‐CoP/Ni_2_P displays the relatively small R_ct_ value (R_ct_ = 4.2 Ω) among all the samples (Figure S21 and Table S8, Supporting Information). As depicted in Figure S22 and Table S9 (Supporting Information), Ru‐CoP/Ni_2_P exhibits a relatively large C_dl_ value of 45.7 mF cm^−2^ in comparison to other samples, suggesting its ECSA value of 1142.5 cm^2^. In addition, Ru‐CoP/Ni_2_P was found to exhibit excellent *j*_ECSA_ as shown in Figure S23 (Supporting Information). The TOF and mass activity of Ru‐CoP/Ni_2_P (1.12 s^−1^ and 2289.5 mA mg_Ru_^−1^) are 8.7 and 16.7 times higher than those of commercial Pt/C, respectively (Figures S24 and S25, Supporting Information). The i‐t curve recorded at 100 mA cm^−2^ for 50 h shows no significant decay (Figure 4f), indicating its excellent stability. Similarly, Figure 4g and Tables S10 and S11 (Supporting Information) present the comparison of the overpotential between Ru‐CoP/Ni_2_P and other previously reported highly active HER catalysts, illustrating the outstanding HER performance of Ru‐CoP/Ni_2_P in neutral and acidic conditions. Above all, the Ru‐CoP/Ni_2_P heterojunction catalyst exhibits remarkable activity and stability for HER over a wide pH range.

**Figure 5:**
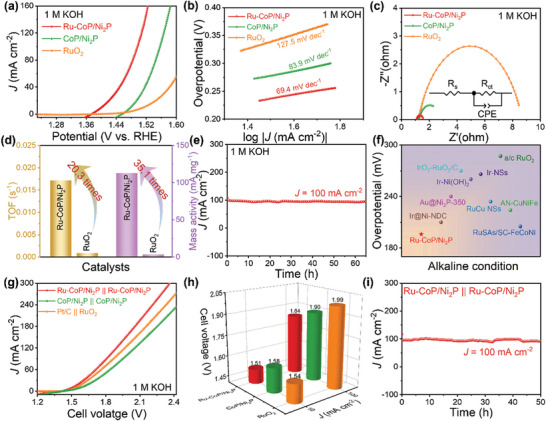
a) The LSV curves of Ru‐CoP/Ni_2_P, CoP/Ni_2_P, and RuO_2_ for OER in 1.0 m KOH. b) The corresponding Tafel slope plots. c) EIS spectra and equivalent circuit diagram. d) Calculated TOF data and mass activity of Ru‐CoP/Ni_2_P and RuO_2_. e) *i*–*t* curve of Ru‐CoP/Ni_2_P at 100 mA cm^−2^ for 65 h. f) Comparison of overpotentials (η_10_) for recently reported advanced OER electrocatalysts. g) The LSV curves of Ru‐CoP/Ni_2_P || Ru‐CoP/Ni_2_P, CoP/Ni_2_P || CoP/Ni_2_P and Pt/C || RuO_2_ for overall water splitting in 1.0 m KOH. h) The comparison of water splitting potential at 10 and 100 mA cm^−2^ for Ru‐CoP/Ni_2_P || Ru‐CoP/Ni_2_P, CoP/Ni_2_P || CoP/Ni_2_P and Pt/C || RuO_2_. i) *i*–*t* curve of Ru‐CoP/Ni_2_P at 100 mA cm^−2^ for 50 h.

In addition to the excellent HER performance of Ru‐CoP/Ni_2_P in all pH values, the OER performance of catalysts also deserves to be explored under alkaline condition. As shown in **Figure** **5**a, the polarization curves of the samples exhibit significantly higher OER activity compared to commercial RuO_2_ and CoP/Ni_2_P. Ru‐CoP/Ni_2_P only needs an overpotential of 171 mV to obtain the current density of 10 mA cm^−2^, which is better than that of CoP/Ni_2_P (248 mV) and RuO_2_ (283 mV). Notably, Ru‐CoP/Ni_2_P requires a low overpotential of 267 mV at 100 mA cm^−2^. Figure 5b clearly shows that Ru‐CoP/Ni_2_P exhibits a Tafel slope of 69.4 mV dec^−1^, which is lower than that of CoP/Ni_2_P (83.9 mV dec^−1^) and RuO_2_ (127.5 mV dec^−1^), indicating its quicker reaction kinetics. The fitted Nyquist plots and equivalent circuit diagram are shown in Figure 5c and Table S12 (Supporting Information), and Ru‐CoP/Ni_2_P displays the fastest electrode kinetics with the smallest R_ct_ value (R_ct_ = 0.5 Ω) among the other catalysts. Moreover, the C_dl_ value of Ru‐CoP/Ni_2_P is 252.5 mF cm^−2^, surpassing that of CoP/Ni_2_P (82 mF cm^−2^) and RuO_2_ (21.3 mF cm^−2^) (Figure S26, Supporting Information). This suggests that Ru‐CoP/Ni_2_P exposes more active sites during the OER process (Table S13, Supporting Information). The TOF and mass activity were compared in Figures S27 and S28 (Supporting Information). Ru‐CoP/Ni_2_P displays the TOF values of 0.017 s^−1^ and mass activity of 112.9 mA mg_Ru+Co_^−1^, which are 20.3 and 35.1 times higher than those of RuO_2_, respectively (Figure 5d). Long‐term durability test (Figure 5e) demonstrates that Ru‐CoP/Ni_2_P displays excellent stability at 100 mA cm^−2^ for 65 h due to the negligible changes of potential. The excellent stability of Ru‐CoP/Ni_2_P is primarily attributed to the interface confinement effect. As shown in Figure 5f and Table S14 (Supporting Information), the overpotential of Ru‐CoP/Ni_2_P at 10 mA cm^−2^ is much lower than that of mostly previous reported literatures, confirming its remarkable activity for oxygen evolution.

**Figure 6:**
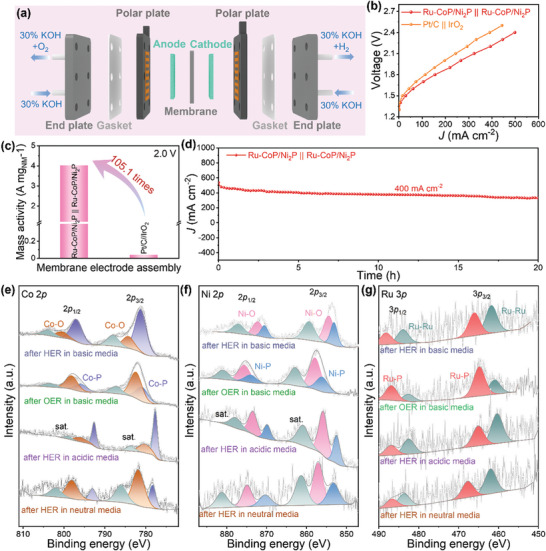
a) Schematic diagram of AWE. b)*I*–*V* curves of Ru‐CoP/Ni_2_P || Ru‐CoP/Ni_2_P and Pt/C || IrO_2_ in AWE. c) The calculated mass activity data of Ru‐CoP/Ni_2_P || Ru‐CoP/Ni_2_P and Pt/C || IrO_2_ at 2.0 V. d) The stability test of Ru‐CoP/Ni_2_P || Ru‐CoP/Ni_2_P system in AWE. High‐resolution XPS spectra of e) Co 2*p*, f) Ni 2*p*, and g) Ru 3*p* after HER and OER.

Based on the above results, Ru‐CoP/Ni_2_P possesses multi‐function catalytic activity for OER and HER. Here, a two‐electrode system was employed to evaluate the OWS performance under alkaline condition. Ru‐CoP/Ni_2_P was used as both the anode and cathode catalysts during testing. As shown in Figure 5g,h, the LSV curves for OWS reveal that Ru‐CoP/Ni_2_P || Ru‐CoP/Ni_2_P only requires a low cell voltage of 1.51 and 1.84 V to reach the current density of 10 and 100 mA cm^−2^, respectively, outperforming CoP/Ni_2_P || CoP/Ni_2_P (1.58 and 1.90 V) and commercial Pt/C || RuO_2_ (1.54 and 1.99 V). The cell voltage at 10 mA cm^−2^ is lower than that of most of reported electrocatalysts (Table S15, Supporting Information), suggesting the superior OWS activity of Ru‐CoP/Ni_2_P system. Additionally, Ru‐CoP/Ni_2_P system demonstrates excellent durability during 50 h chronoamperometry test at 100 mA cm^−2^ due to the slightly degradation of cell voltage (Figure 5i). Furthermore, the AWE device was applied to evaluate the potential industrial application of Ru‐CoP/Ni_2_P. The schematic diagram of AWE is shown in **Figure** **6**a. Ru‐CoP/Ni_2_P was used as anode and cathode catalysts, and current‐voltage (*I*–*V*) curves were recorded in 30% KOH solution at 80 °C. Figure 6b directly reveals Ru‐CoP/Ni_2_P || Ru‐CoP/Ni_2_P system only requires 1.8 and 2.1 V to reach the current density of 155 and 324 mA cm^−2^, which is superior than commercial Pt/C || IrO_2_ (113 and 244 mA cm^−2^). The mass activity was calculated based on the actual mass loading of noble metal. Ru‐CoP/Ni_2_P || Ru‐CoP/Ni_2_P system displays outstanding mass activity compared with Pt/C || IrO_2_. In detail, the mass activity of Ru‐CoP/Ni_2_P || Ru‐CoP/Ni_2_P system is 4017 mA mg_NM_^−1^ at 2.0 V (Figure 6c), which is 105.1 times higher than that of Pt/C || IrO_2_ (38 mA mg_NM_^−1^). Furthermore, the long‐term stability was tested at 400 mA cm^−2^ for 20 h with negligible attenuation (Figure 6d). It is proved that Ru‐CoP/Ni_2_P displays superior activity and stability, exhibiting great application potential.

The XPS of Ru‐CoP/Ni_2_P post HER and OER testing were recorded to analyze the composition changes. As shown in Figure 6f–h and Figure S29 (Supporting Information), the high‐resolution spectra of Co 2*p* and Ni 2*p* still show peaks of Co/Ni─O and Co/Ni─P and the bonds of Ru─Ru and Ru─P are still present in the high‐resolution Ru 3*p* spectra, which is similar to the initial spectra, indicating no obvious composition and structural changes after the test. Compared to the initial Ru‐CoP/Ni_2_P, the content of Co─P and Ni─P increased after the testing, suggesting the superior stability of the catalysts.^[^ ^41^ ^]^ Notably, the increased electron density around Ru shows the higher valence state of Ru, which drives from partial surface oxidation of Ru‐CoP/Ni_2_P during the OER process and confirms the superior electrochemical activity.^[^ ^39^ ^]^ Moreover, the morphology and phase of Ru‐CoP/Ni_2_P after OER were characterized to explore its structural stability. XRD pattern displays the existence of CoP and Ni_2_P without Ru species, which reveals the retained compositions of Ru‐CoP/Ni_2_P (Figure S30, Supporting Information). SEM images suggest that Ru‐CoP/Ni_2_P maintained the nanoneedle structure with tip‐twisted feature (Figure S31, Supporting Information). Besides, nanoneedles and hetero‐interface structure still can be observed from TEM and HRTEM images, demonstrating the structural stability owing to the almost unchanged morphology (Figure S32, Supporting Information). EDS elemental mapping images show the uniformly dispersed of each element. Therefore, Ru‐CoP/Ni_2_P displayed superior structural stability and showed great potential of industrial application.

### 2.3 Mechanism Studies

**Figure 7:**
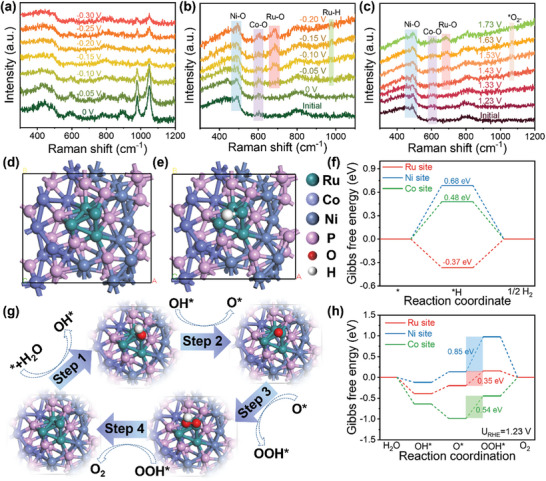
a) In situ Raman spectra of Ru‐CoP/Ni_2_P recorded in 0.5 m H_2_SO_4_ from 0 to −0.30 V (vs RHE). b) In situ Raman spectra of Ru‐CoP/Ni_2_P recorded in 1.0 m KOH from 0 to −0.20 V (vs RHE). c) In situ Raman spectra of Ru‐CoP/Ni_2_P recorded in 1.0 m KOH from 1.23 to 1.73 V (vs RHE). d) DFT‐optimized model of Ru‐CoP/Ni_2_P. e) The schematic diagram of absorbed H atoms at Ru site. f) Gibbs free energy on different sites of Ru‐CoP/Ni_2_P for HER under alkaline condition. g) The schematic diagram of alkaline OER elemental steps at Ru site. h) Gibbs free energy on different sites of Ru‐CoP/Ni_2_P for OER under alkaline condition.

In order to study the electrochemical reaction process, in situ Raman was carried out. **Figure** **7**a displays the in situ Raman spectra in the process of HER in 0.5 m H_2_SO_4_ electrolyte from 0 to −0.30 V (vs RHE). The unchanged peaks suggest the stability of Ru‐CoP/Ni_2_P in acidic condition. Furthermore, the in situ Raman spectra were measured in 1.0 m KOH toward HER and OER (Figure 7b). The newly formed Ru‐O bond located at ≈685 cm^−1^ suggests the generation of Ru‐OH* intermediates, which derives from the cleavage of H_2_O molecules.^[^ ^42^, ^43^ ^]^ During HER, the appearance of Ru‐H (≈980 cm^−1^) after the applied potential reached −0.05 V strongly imply the formation of Ru─H* intermediates, indicating that Ru atoms act as the main active sites (Figure 7b).^[^ ^44^, ^45^ ^]^ For OER process, the vibration of superoxide (*O_2_^‐^) was detected (≈1070 cm^−1^), which can be ascribed to the intermediates in the adsorption evolution mechanism (AEM) (Figure 7c).^[^ ^46^, ^47^ ^]^ The *O_2_^‐^ and *O_2_^2‐^ are regarded as the characteristic species of AEM and lattice oxygen mechanism (LOM), respectively. The *O_2_^2‐^ can bind with tetramethylammonium cations (TMA^+^) and lead to a significant decrease of OER activity via LOM. Thus, the LSV curves are compared before and after adding TMA^+^ during OER testing. As shown in Figure S33 (Supporting Information), the OER activity of Ru‐CoP/Ni_2_P was only slightly changed after the addition of 10 mm TMA^+^ to the 1 m KOH electrolyte, confirming the AEM pathway of OER for Ru‐CoP/Ni_2_P. To unravel the main active sites responsible for the improved catalytic activity, DFT calculations were performed toward HER and OER in alkaline condition. The constructed model of Ru‐CoP/Ni_2_P is shown in Figure 7d with the hetero‐interface of CoP (001) and Ni_2_P (100). The four Ru atoms with Ru─Ru and Ru─P bonds were anchored on the hetero‐interface, which is consistent with EXAFS fitting results. The Ru, Co, and Ni sites were selected to explore the adsorption and desorption of reaction intermediates. Figure 7e and Figures S34 and S35 (Supporting Information) display the adsorption of H* on Ru, Co, and Ni sites in the process of HER. As shown in Figure 7f, the rate‐determine step (RDS) of HER at Co and Ni sites is the adsorption of H*, while the RDS at Ru site is the desorption of H_2_. The Ru site displays a lower Gibbs free energy (ΔG_H*_) (−0.37 eV) compared with Co site (0.48 eV) and Ni site (0.68 eV), closing thermo‐neutral condition required for an ideal HER. It is proved that Ru sites act as the active sites during HER, which is consistent with the results of in situ Raman spectra. The OER process on Ru, Co, and Ni sites of Ru‐CoP/Ni_2_P are shown in Figure 7g and Figures S36 and S37 (Supporting Information), involving the adsorption and desorption processes of OH*, O*, and OOH* intermediates. As depicted in Figure 7h, the RDS of OER at three sites is the conversion from O* to OOH*. The ΔG_H*_ value of Ru site (0.35 eV) is comparatively lower than that of Ni site (0.85 eV) and Co site (0.54 eV), indicating that the Ru site serves as the optimal active center for OER in Ru‐CoP/Ni_2_P and contributes to the formation of O─O bond. The facilitation of the O* to OOH* transition leads to rapid OER reaction kinetics. Above all, Ru atoms are conducive to the adsorption and desorption of intermediates, which are the real active site for alkaline HER and OER.

## 3 Conclusion

In summary, Ru‐CoP/Ni_2_P is synthesized with the anchored Ru clusters on cobalt and nickel bimetallic phosphide heterojunctions. Benefitting from the interface synergy and confinement effect, Ru‐CoP/Ni_2_P achieves highly efficient HER and OER performance with low overpotentials of 53, 64, and 125 mV at 10 mA cm^−2^ under acidic, alkaline, and neutral conditions, respectively. Meanwhile, it just requires 171 mV to reach the current density of 10 mA cm^−2^ for alkaline OER activity, and the corresponding TOF and mass activity are 20.3 and 35.1 times higher than those of RuO_2_. Moreover, the assembled Ru‐CoP/Ni_2_P || Ru‐CoP/Ni_2_P system shows excellent activity with 1.51 V of cell voltage at 10 mA cm^−2^ for OWS and superior mass activity of 4017 mA mg_NM_^−1^ at 2.0 V for AWE, which is 105.1 times higher than that of Pt/C || IrO_2_. Further mechanism studies reveal that Ru atoms serve as catalytic active sites, contributing to the desorption of H_2_ for HER and promoting the transformation of O* to OOH* for OER. This work provides a feasible approach to fabricate heterogeneous‐interface materials and emphasizes the importance of interface confinement effect.

## Conflict of Interest

The authors declare no conflict of interest.

## Supporting information

Supporting Information

## Data Availability

The data that support the findings of this study are available from the corresponding author upon reasonable request.
