# Supplementary material for: Constructing CoP/Ni2P Heterostructure Confined Ru Sub‐Nanoclusters for Enhanced Water Splitting in Wide pH Conditions
Source: Adv Sci (Weinh). 2024 Jul 11;11(35):2401398. doi: 10.1002/advs.202401398 (PMC11425266; doi:10.1002/advs.202401398)
Supplement: Supplementary file 1 — Supporting Information [file ADVS-11-2401398-s001.docx]

Supporting Information

**Constructing CoP/Ni_2_P Heterostructure Confined Ru Sub-Nanoclusters for Enhanced Water Splitting in Wide pH Conditions**

*Huimin Zhang^1^, Wenhao Liu^1^, Zhenhao Li^2^, Liang Qiao^2^, Kebin Chi^2^, Xiaoyan Guo^1^, Dong Cao^1*^, Daojian Cheng^1*^*

^1^State Key Laboratory of Organic-Inorganic Composites, College of Chemical Engineering, Beijing University of Chemical Technology, Beijing 100029, People’s Republic of China

^2^PetroChina Petrochemical Research Institute, Beijing 102206, China

^*^To whom correspondence should be addressed. E-mail: [chengdj@mail.buct.edu.cn](mailto:chengdj@mail.buct.edu.cn), caod@mail.buct.edu.cn.

**Experimental section**

**Chemicals**

All chemicals used in this study were purchased from reputable suppliers and used without further purification. Cobalt nitrate hexahydrate (Co(NO_3_)_2_·6H_2_O, 99%), urea (CO(NH_2_)_2_, 99%) and sodium hypophosphite monohydrate (NaH_2_PO_2_·H_2_O, 99%), potassium hydroxide (KOH, 95%), ruthenium chloride (RuCl_3_, AR), and Pt/C (20 wt% Pt on Vulcan XC-72R) were obtained from Macklin. Nickel foam with a thickness of 0.5 mm was purchased from Yujiang Hardware, while ammonium fluoride (NH_4_F, AR 98%) was purchased from General-reagent. Ethanol (99.7%) was sourced from Beijing Chemical Works. The deionized water used in this study was purified to ensure a high level of purity (> 18.25 MΩ cm^-1^) from a purification system.

**Synthesis of Ru-Doped Co-Ni Bimetallic Phosphide Nanoneedles (Ru-CoP/Ni_2_P)**

To prepare the Ru-CoP/Ni_2_P catalyst, 2 mmol of Co(NO_3_)_2_·6H_2_O, 5 mmol of a NH_4_F, and 10 mmol of urea were dissolved in 35 mL deionized water. Then the mixed solution was transferred into a 50 mL Teflon-lined autoclave, and a 2×5 cm piece of nickel foam was immersed in the solution. The autoclave was then placed in a vacuum oven and heated to 120°C for 6 hours. After naturally cooling to room temperature, the nickel foam with catalyst was washed by ethanol and dried at 40°C overnight.

Subsequently, 0.02 mmol RuCl_3_ was dissolved in 30 mL of deionized water. The dried nickel foam with catalyst was soaked in the solution and stirred magnetically for 24 hours. The nickel foam was then dried and subjected to low-temperature phosphating calcination. The sample was heated with a rate of 3°C/min to 350°C and maintained for 2 hours under nitrogen atmosphere. Excess NaH_2_PO_2_·H_2_O was applied as phosphorus source to ensure that the nickel in the foam nickel could be phosphatized. After cooling to room temperature, the desired Ru-CoP/Ni_2_P catalyst was obtained.

**Synthesis of Co-Ni Bimetallic Phosphide Nanoneedles (CoP/Ni_2_P)**

The synthesis procedure of CoP/Ni_2_P was similar to that of Ru-CoP/Ni_2_P, with the only difference being that the absence of an impregnation step in RuCl_3_ solution. After phosphating in tube furnace, the interface catalyst CoP/Ni_2_P is obtained.

**Synthesis of Nickel Phosphide (Ni_2_P) and Cobalt Phosphide (CoP)**

The nickel foam was placed directly in a ceramic boat downstream of the tube furnace. After cooling to room temperature, the sample Ni_2_P is obtained. The synthesis procedure of CoP was similar to that of CoP/Ni_2_P, except that no nickel foam was placed in autoclave. Then, same phosphating condition was used to obtain CoP.

**Materials Characterization**

X-ray diffraction (XRD) data were collected by a Rigaku D/Max 2550 diffractometer with Cu Kα radiation (λ=1.5418 Å). Scanning electron microscope (SEM) characterization was performed on a HITACHI S-4800 at an accelerating voltage of 20 kV. Besides, a ThermoFisher-ESCALab250 with Al-Kα X-ray radiation (1486.6.6 eV) was applied to obtain the X-ray photoelectron spectroscopy (XPS) data. Transmission electron microscope (TEM), energy dispersive X-ray spectroscopy (EDX), high-resolution TEM (HRTEM) and high-angle annular dark-field scanning TEM (HAADF-STEM) images were characterized by a Tecnai G2 F20 S-Twin HRTEM with an accelerating voltage of 200kV. Electron paramagnetic resonance (EPR) was measured on a Bruker EMX PLUS in 3.170 mW. Inductive Coupled Plasma Emission Spectrometer (ICP) was carried out by using the Agilent 725ES (OES). The X-ray absorption find structure spectra (Ru *K-*edge) were collected at 1W1B station in Beijing Synchrotron Radiation Facility (BSRF). The storage rings of BSRF was operated at 2.5 GeV with an average current of 250 mA. In-situ Raman spectroscopy were collected on a confocal microscope Raman system with a 514-nm laser (inVia Reflex). Raman frequency was calibrated by a Si wafer (520 cm^-1^) befroe testing.

**Electrochemical measurement**

All electrochemical measurements were carried out by CHI 760E (CHENHUA, China) electrochemical workstation. The electrochemical performance was performed in an electrolytic cell using a standard three-electrode system at room temperature. The catalyst-loaded nickel foam was cut into 1×2 cm^2^ and clamped by an electrode clip. 1 cm^2^ of nickel foam was immersed in the electrolyte as the working electrode. Saturated calomel electrode (SCE) and graphite rods were applied as reference electrode and counter electrode, respectively. 1 M KOH and 0.5 M H_2_SO_4_ solutions were used as the electrolyte. Cyclic voltammograms (CVs) and linear sweep voltammetry (LSV) was recorded at a scan rate of 100 and 5 mV s^-1^, respectively. All reported potentials are calibrated to the reversible hydrogen electrode (RHE) by the electrochemical equation of E_(RHE)_ = E_(SCE)_ + 0.059 V × pH + 0.242 V. Electrochemical impedance measurement (EIS) was performed at the open-circuit voltage at a frequency range of 100 kHz to 0.01 Hz. The Nyquist plot was fitted by Z-view software.

The electrochemical double-layer capacitance (C_dl_) was calculated from cyclic voltammetry (CV) curves at different scan rates (20-120 mV s^-1^) in the non-Faraday efficiency region:

C_dl_=*J*/v

And the electrochemical active surface area (ECSA) of materials was calculated:

ECSA=C_dl_/C_s_

C_s_ is the specific capacitance of 0.04 mF cm^-2^.

Tafel slope was calculated by the following equation:

η=a+blog(|j|)

Mass activity was calculated by the following equation:^[1]^

*J*_m_=*J*×A/m

For HER, where *J* (mA cm^-2^) is the measured current density, A (1.0 cm^-2^) is the geometry surface area of working electrode immersing in the electrolyte, m is the mass of noble metal (Ru) on the electrode.

For OER, where *J* (mA cm^-2^) is the measured current density, A (1.0 cm^-2^) is the geometry surface area of working electrode immersing in the electrolyte, m is the mass of metal (Ru and Co) on the electrode.

Turnover frequency (TOF) for HER was calculated using the following equation:^[2]^

TOF=I/2nF

where the number 2 means 2 electrons mol^-1^ of H_2_ for HER, I is the current (A) at corresponding overpotential during LSV, F is Faraday constant (96485 C mol^-1^), n is the molar mass of Ru on the electrode surface.

For OER, TOF was calculated by the equation:

TOF=I/4nF

where the number 4 is the number of transferred electron based on that four electrons are required to form one O_2_ molecule.

**XAFS analysis**

The acquired EXAFS data were processed according to the standard procedures using the ATHENA module implemented in the IFEFFIT software packages. The k^3^-weighted EXAFS spectra were obtained by subtracting the post-edge background from the overall absorption and then normalizing with respect to the edge-jump step. Subsequently, k^3^-weighted χ(k) data of Pt L-edge were Fourier transformed to real (R) space using a hanning windows (dk=1.0 Å^-1^) to separate the EXAFS contributions from different coordination shells. To obtain the quantitative structural parameters around central atoms, least-squares curve parameter fitting was performed using the ARTEMIS module of IFEFFIT software packages.

**DFT calculations**

All the calculations are performed in the framework of the density functional theory with the projector augmented plane-wave method, as implemented in the Vienna ab initio simulation package. The generalized gradient approximation proposed by Perdew, Burke, and Ernzerhof is selected for the exchange-correlation potential. The Grimme D3 correction using a coordination number dependent dispersion correction. The cut-off energy for plane wave is set to 450 eV. The energy criterion is set to 10^−4^ eV in iterative solution of the Kohn-Sham equation. A vacuum layer of 15 Å is added perpendicular to the sheet to avoid artificial interaction between periodic images. The Brillouin zone integration is performed using a 2×2×1 k-mesh. All the structures are relaxed until the residual forces on the atoms have declined to less than 0.03 eV/Å.

The Free energy changes (ΔG) of reaction intermediates could be calculated by the following:

ΔG = ΔE + ΔE_ZPE_−TΔS

where ΔE is the adsorption energy on the cluster surface from DFT calculations. The ΔE_ZPE_ and ΔS are the difference for the zero-point energy and entropy. The zero-point energy and entropy are calculated at the standard conditions corresponding to the pressure of 101325 Pa (~1 bar) of H_2_ at the temperature of 298.15 K.


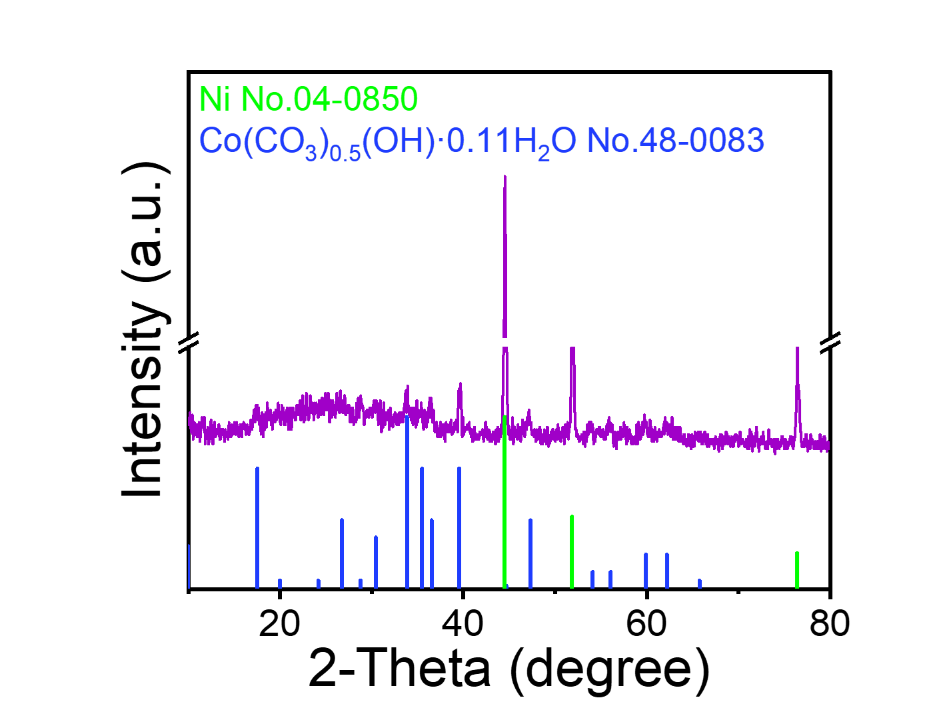


Figure S1. XRD pattern of the sample before phosphating.


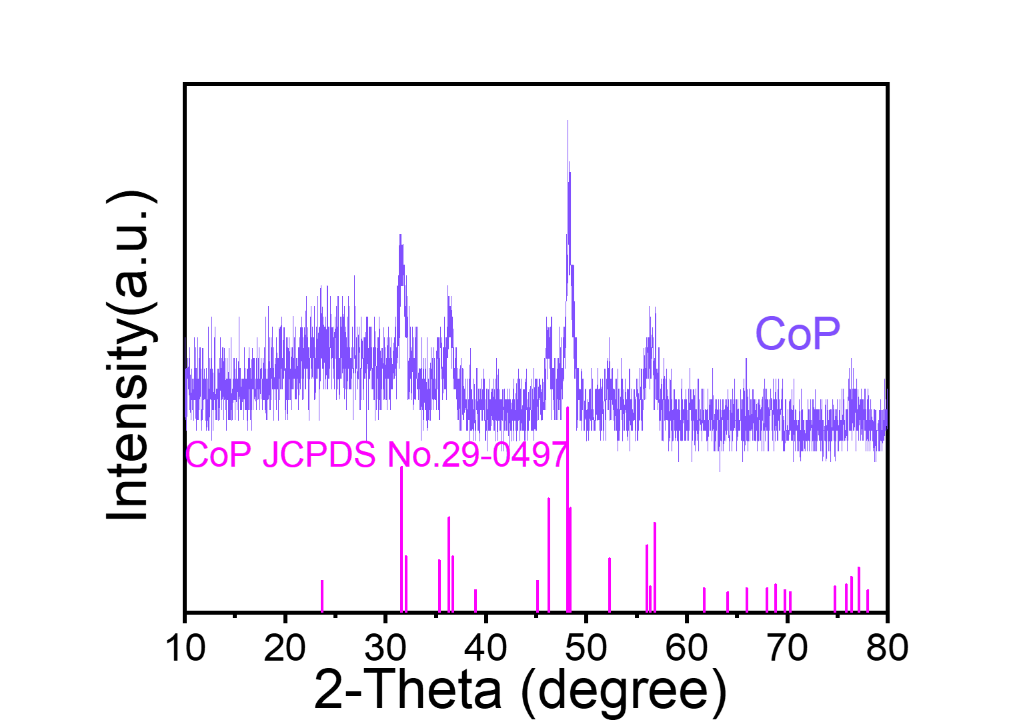


Figure S2. XRD pattern of CoP.


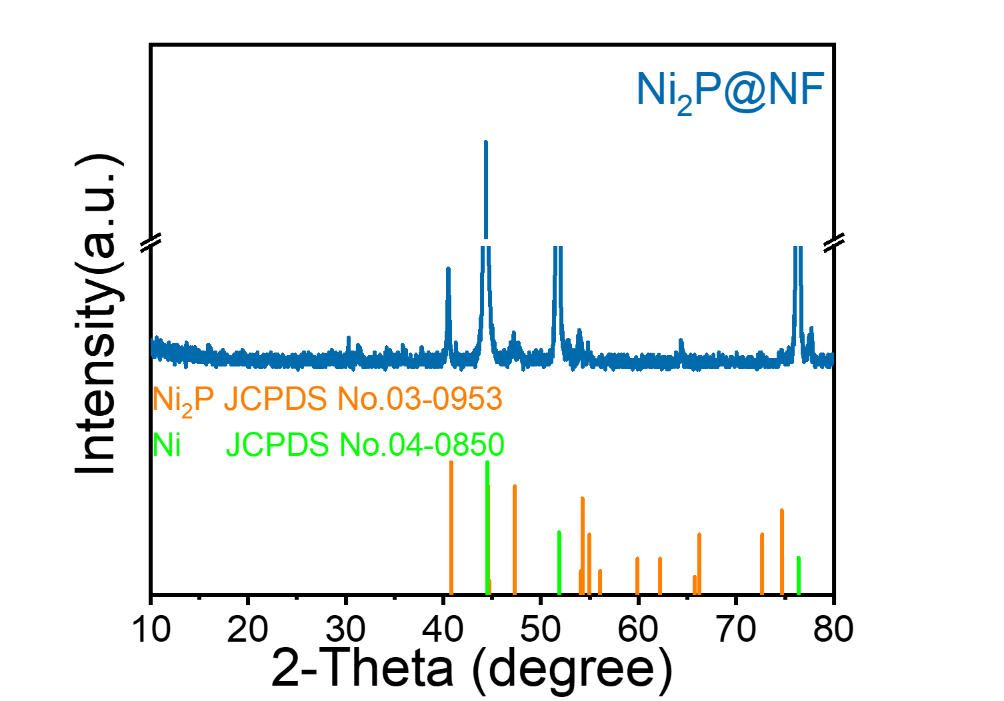


Figure S3. XRD pattern of Ni_2_P.


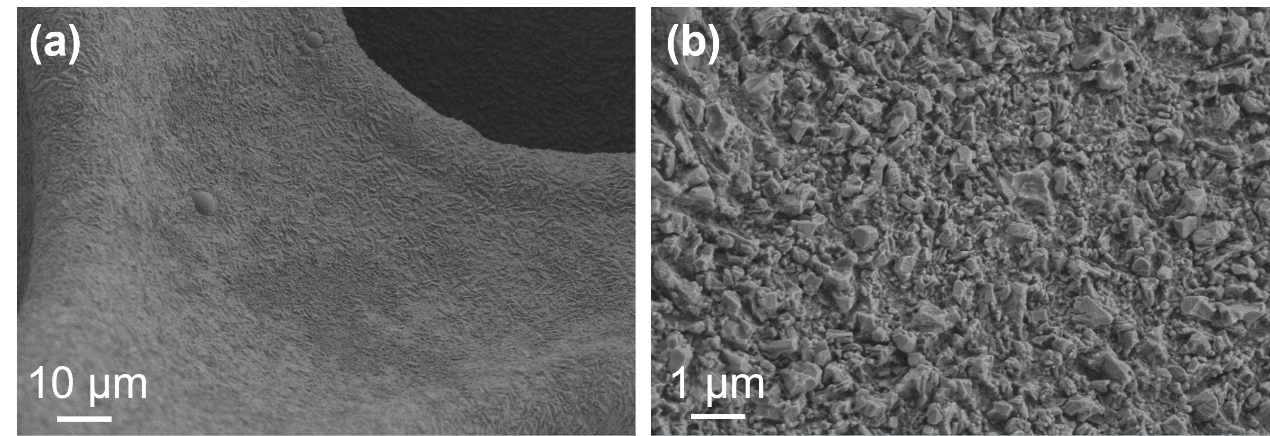


Figure S4. SEM images of Ni_2_P with scale bar of (a) 10 μm and (b) 1 μm.


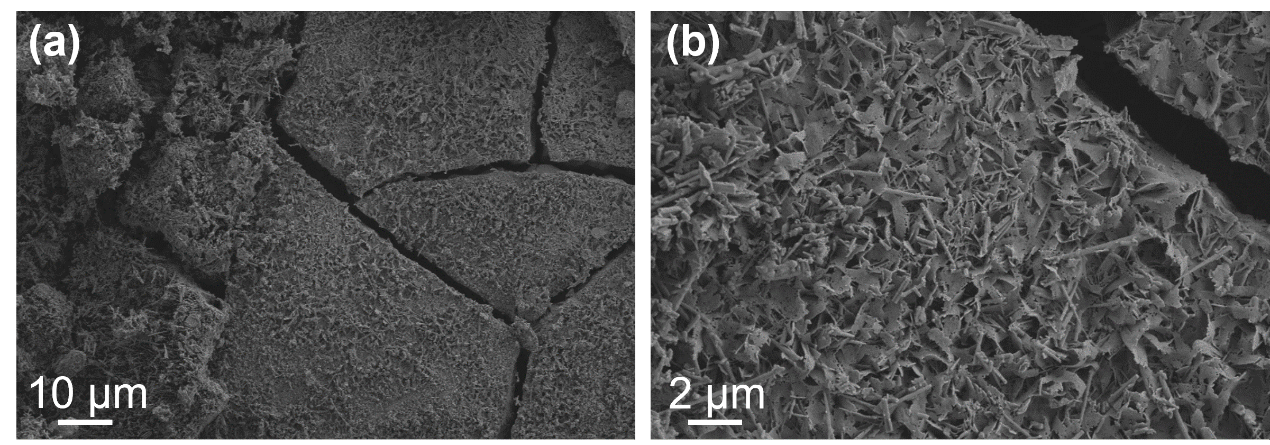


Figure S5. SEM images of CoP with scale bar of (a) 10 μm and (b) 2 μm.


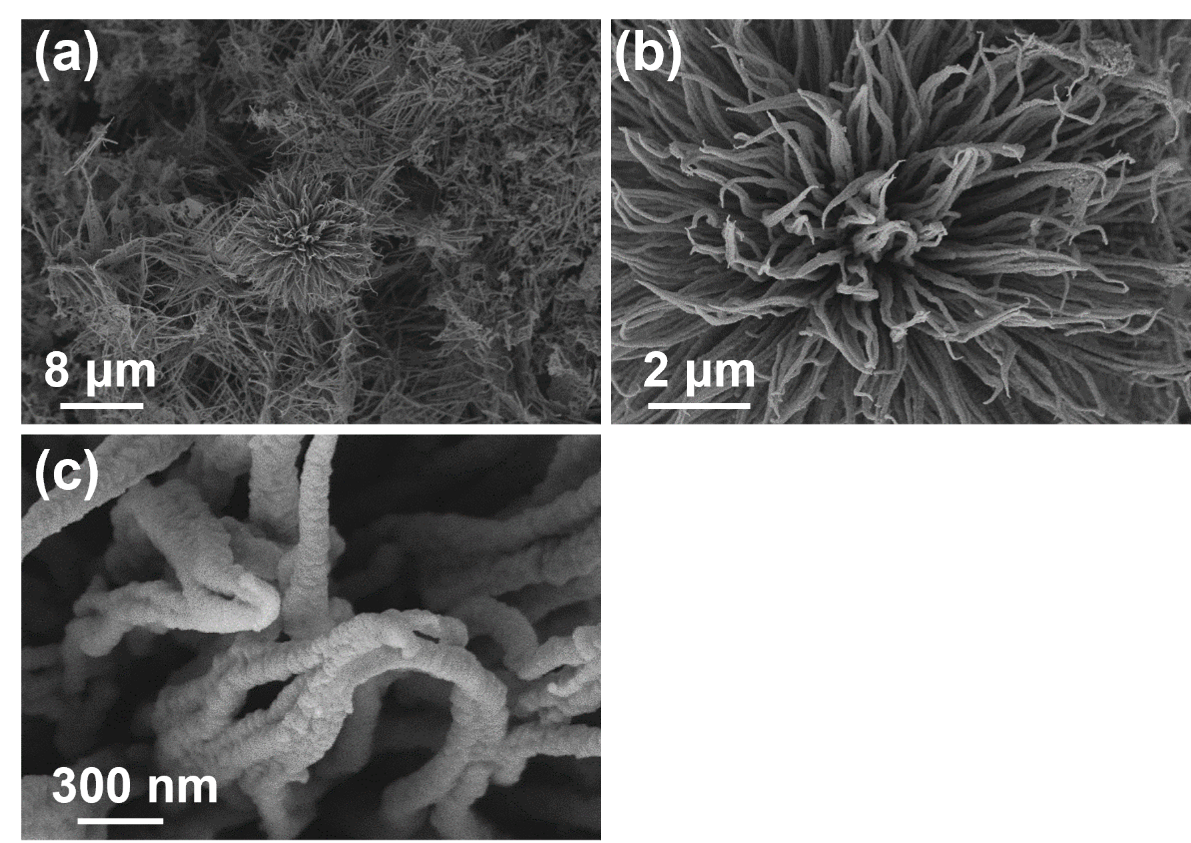


Figure S6. SEM images of Ru-CoP/Ni_2_P with scale bar of (a) 8 μm, (b) 2 μm, (c) 300 nm.


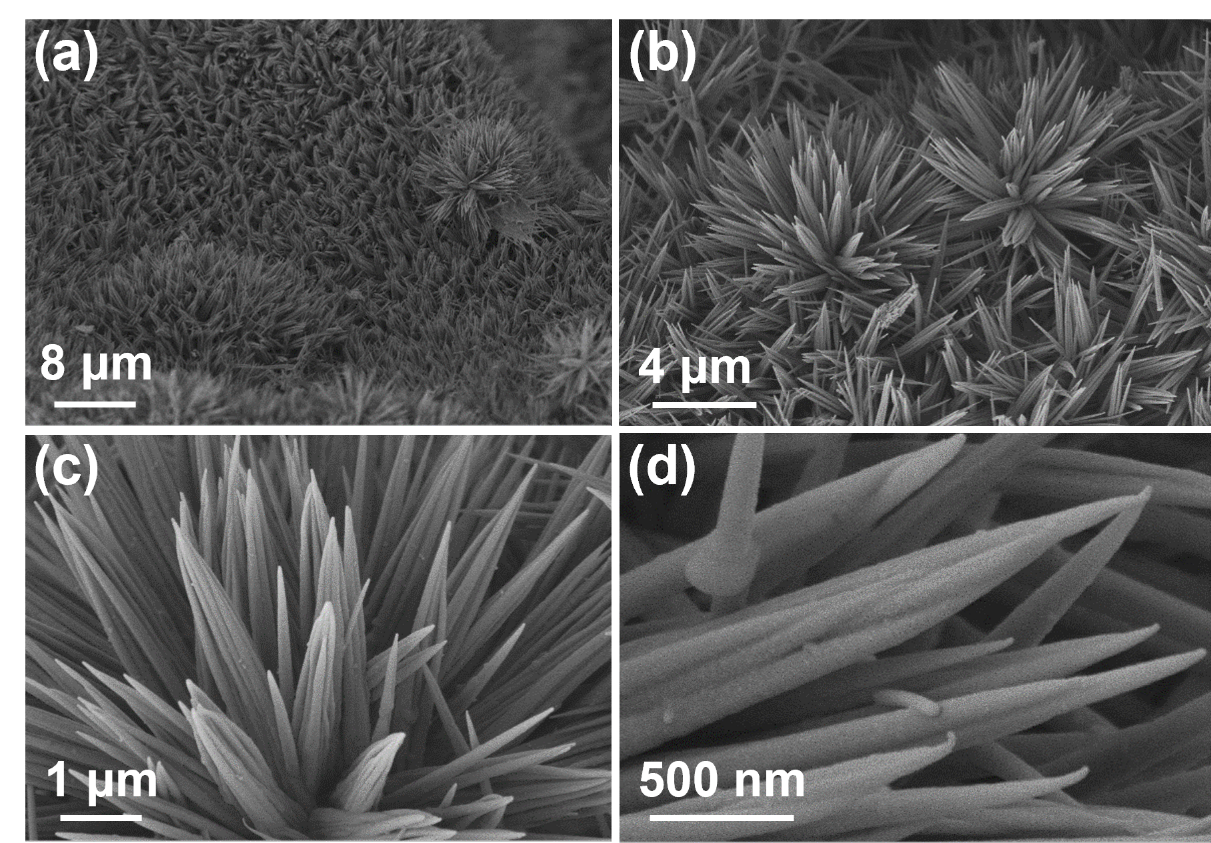


Figure S7. SEM images of CoP/Ni_2_P with scale bar of (a) 10 μm, (b) 4 μm, (c) 1 μm, and (d) 500 nm.


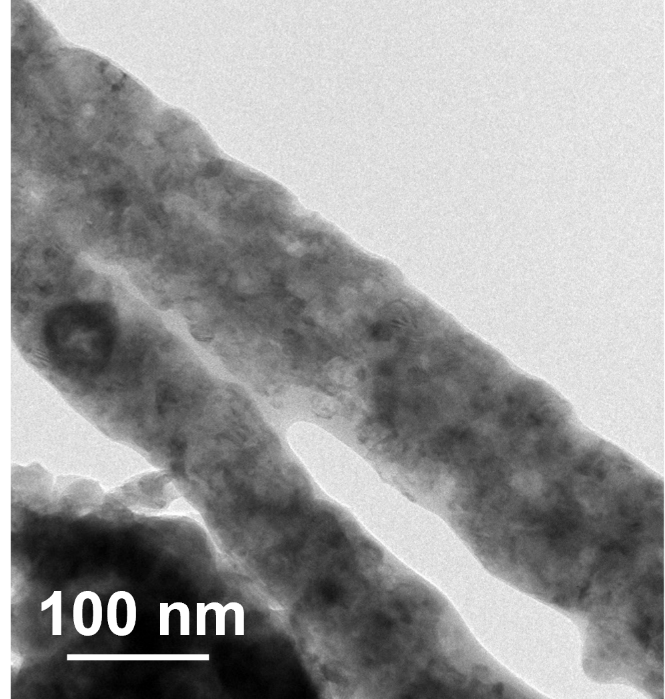


Figure S8. TEM image of Ru-CoP/Ni_2_P.


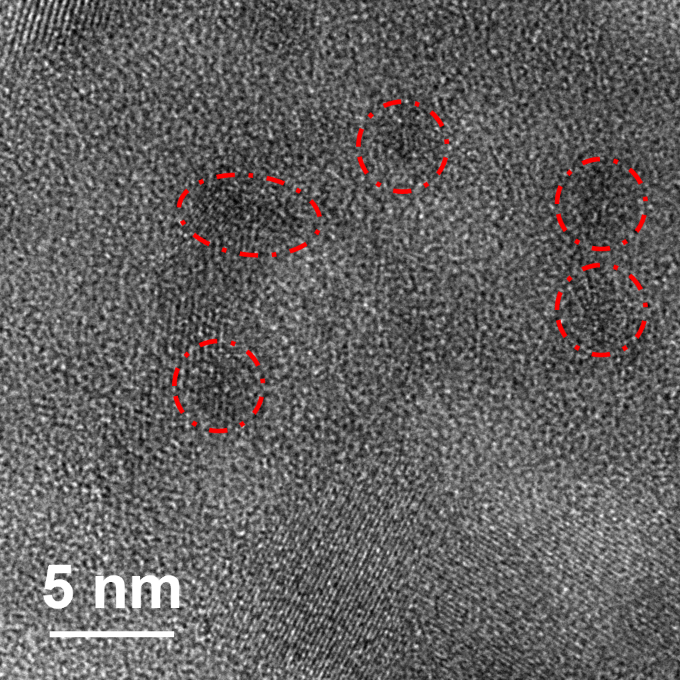


Figure S9. HRTEM image of Ru-CoP/Ni_2_P with the Ru clusters marked by red circles.


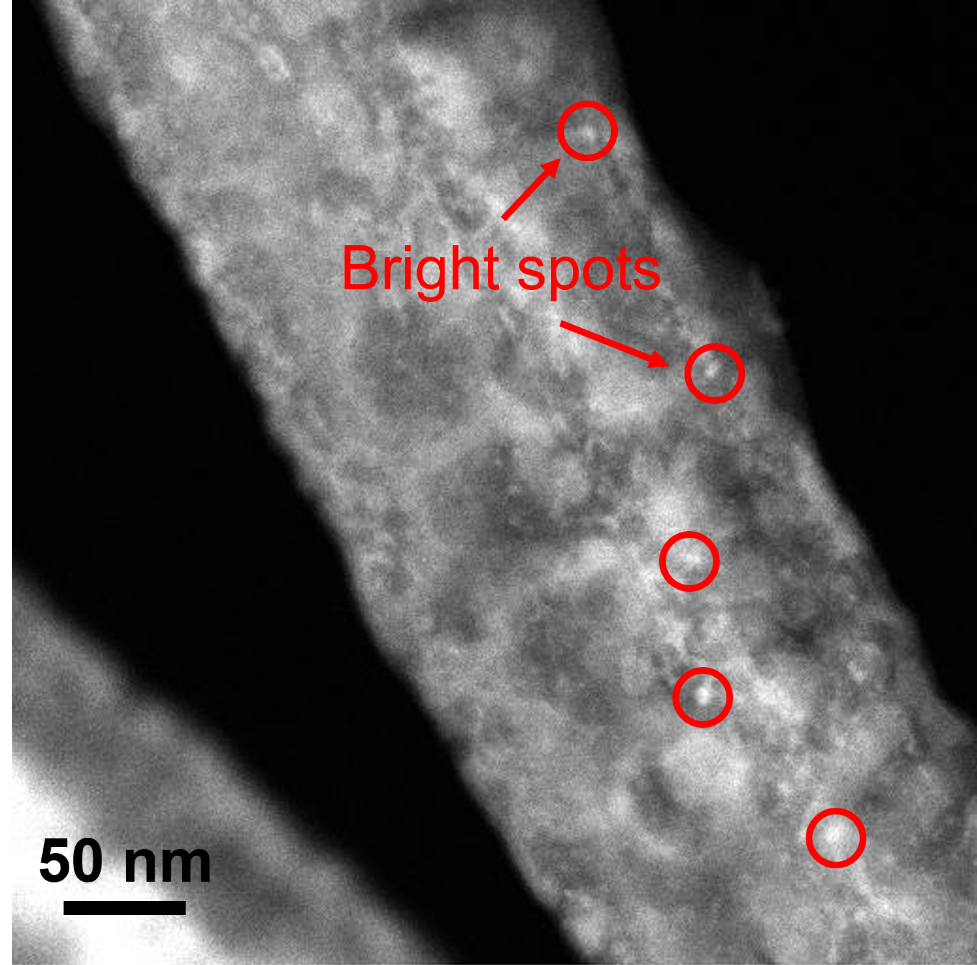


Figure S10. HAADF-STEM image of Ru-CoP/Ni_2_P with scale bar of 50 nm.


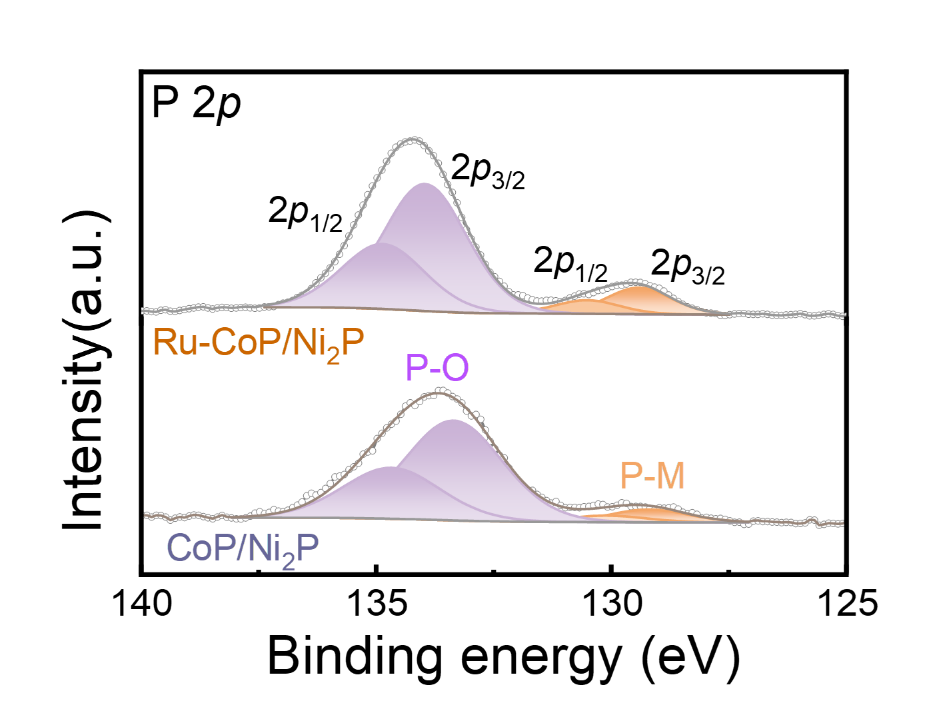


Figure S11. High-resolution P 2*p* spectra of Ru-CoP/Ni_2_P and CoP/Ni_2_P.


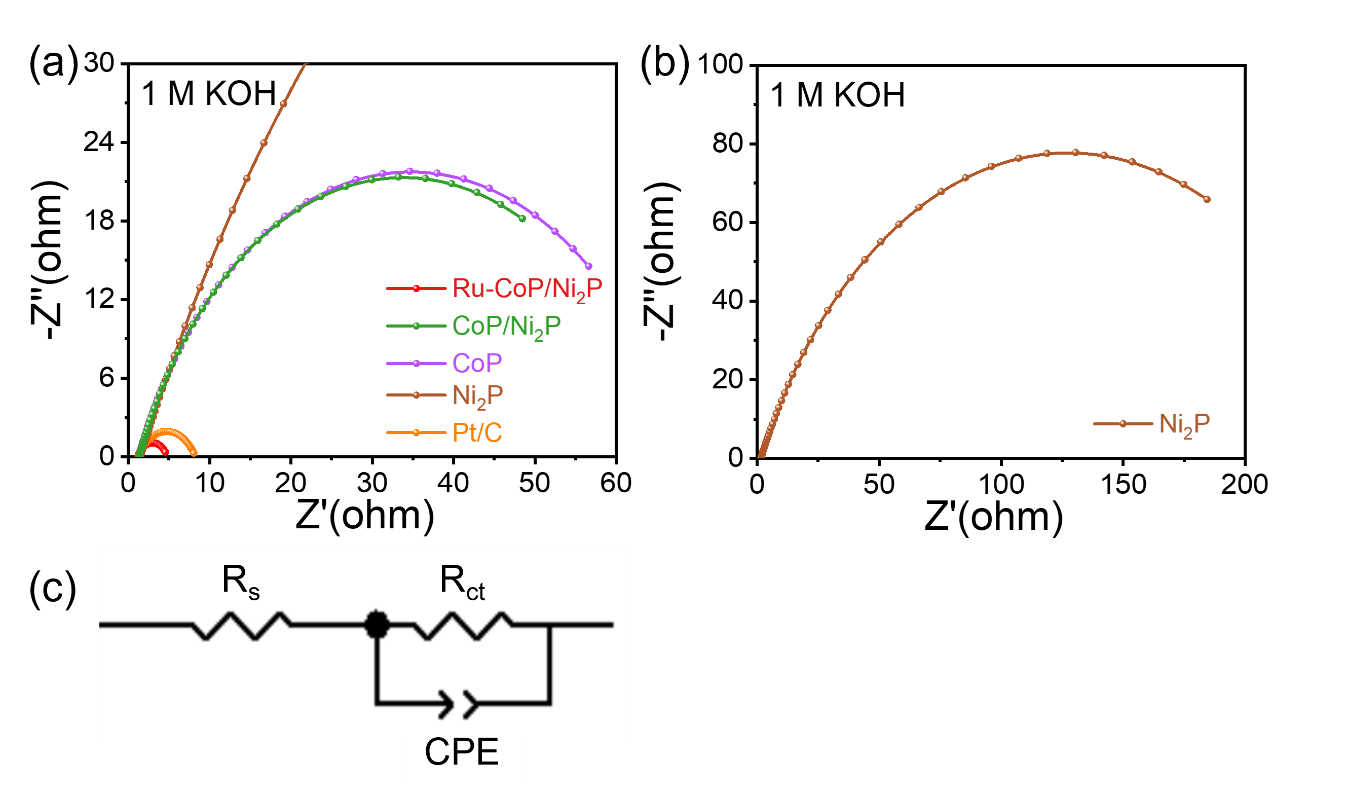


Figure S12. (a, b) EIS spectra of the samples toward HER in alkaline condition. (c) The equivalent circuit diagram.


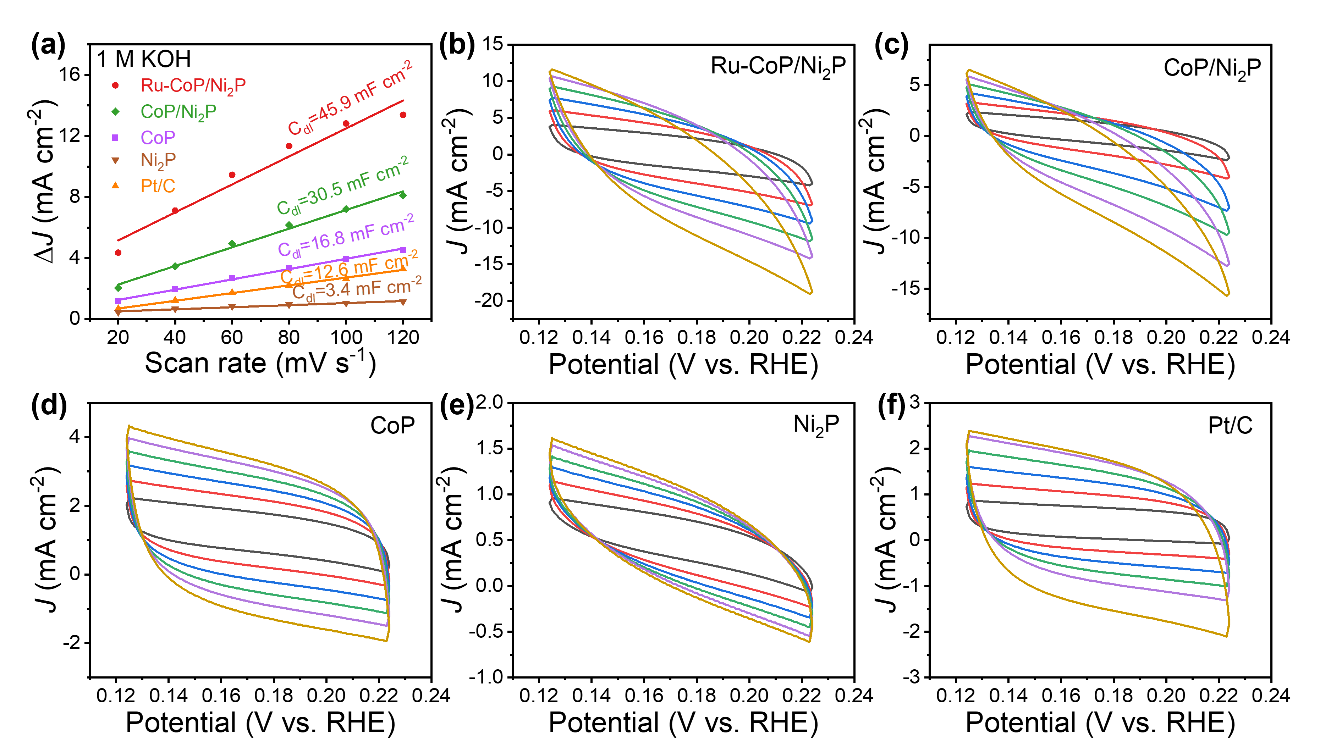


Figure S13. (a) C_dl_ curves of the samples for HER in 1.0 M KOH electrolyte. CVs for (b) Ru-CoP/Ni_2_P, (c) CoP/Ni_2_P, (d) CoP, (e) Ni_2_P, (f) Pt/C, respectively.


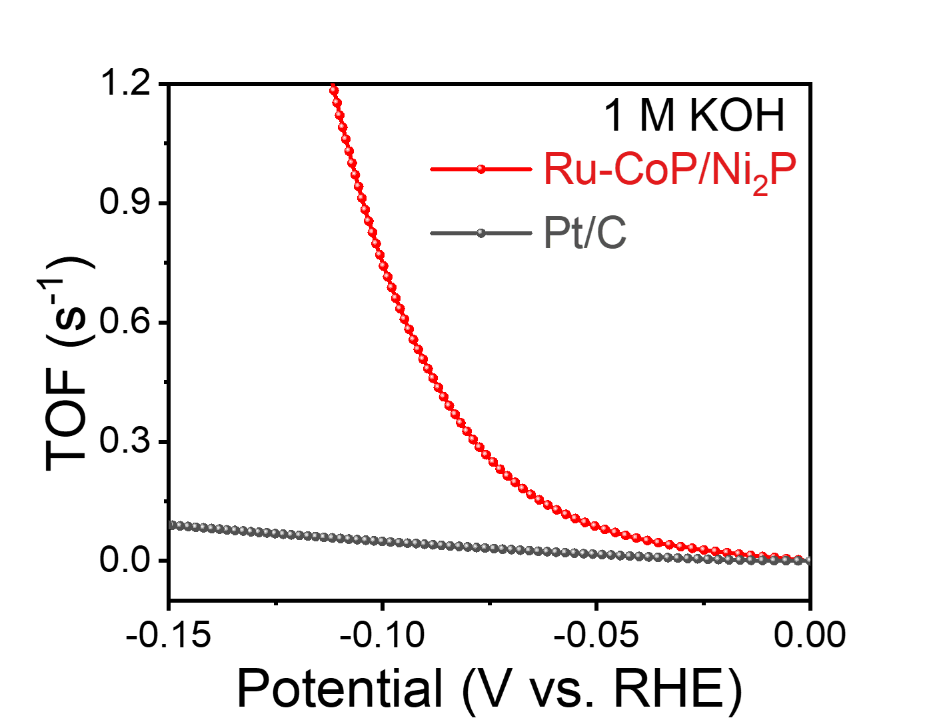


Figure S14. TOF curves of Ru-CoP/Ni_2_P and Pt/C for HER under alkaline condition.


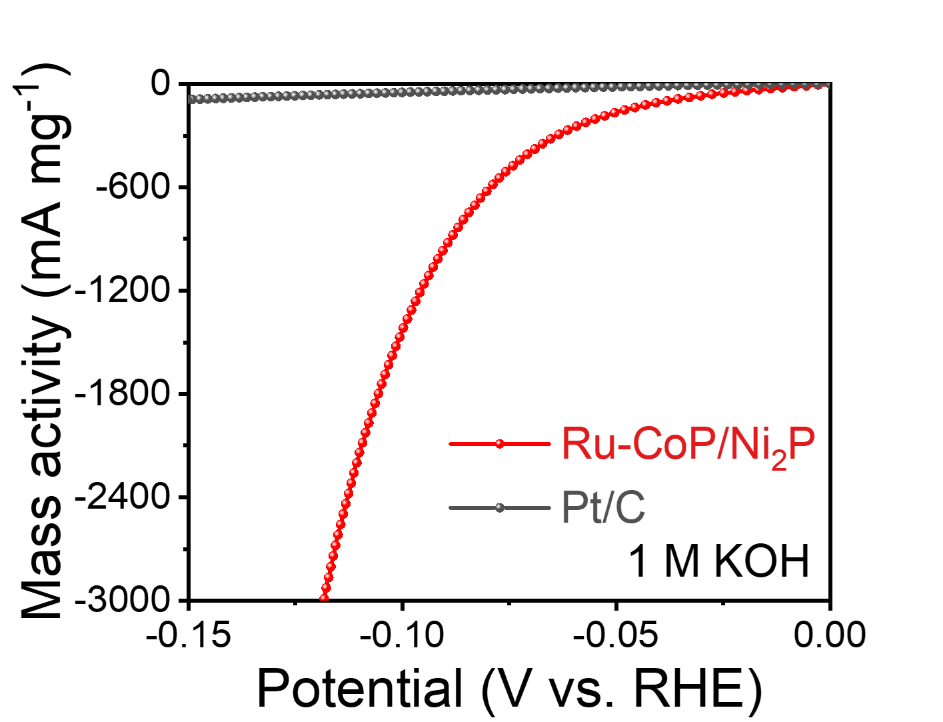


Figure S15. Mass activity plots of Ru-CoP/Ni_2_P and Pt/C for HER under alkaline condition.


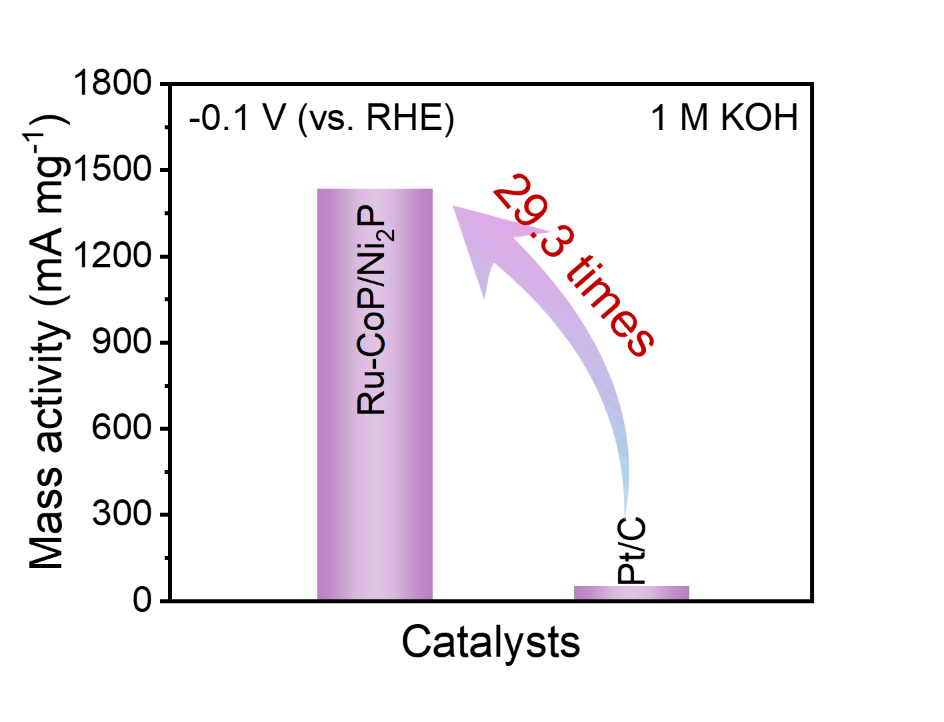


Figure S16. Calculated mass activity of Ru-CoP/Ni_2_P and Pt/C at -0.1 V (vs. RHE) for HER under alkaline condition.


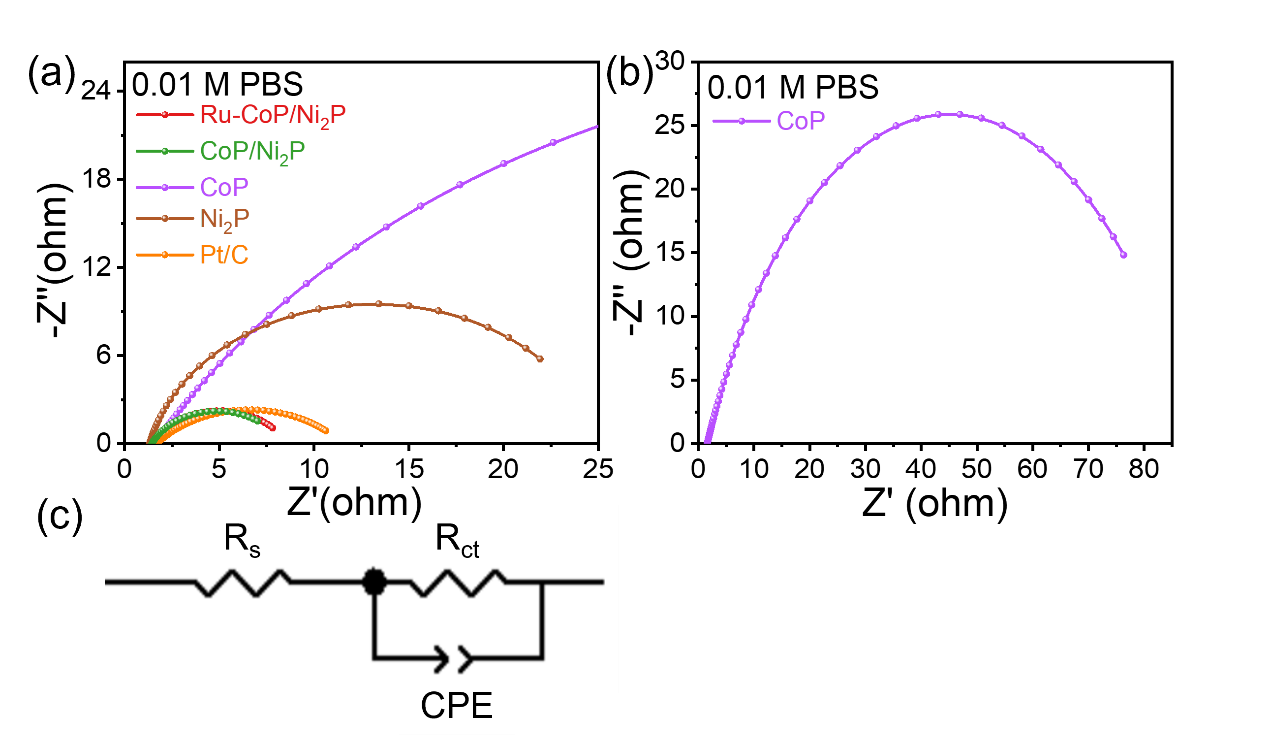


Figure S17. (a, b) EIS spectra of the samples toward HER in neutral condition. (c) The equivalent circuit diagram.


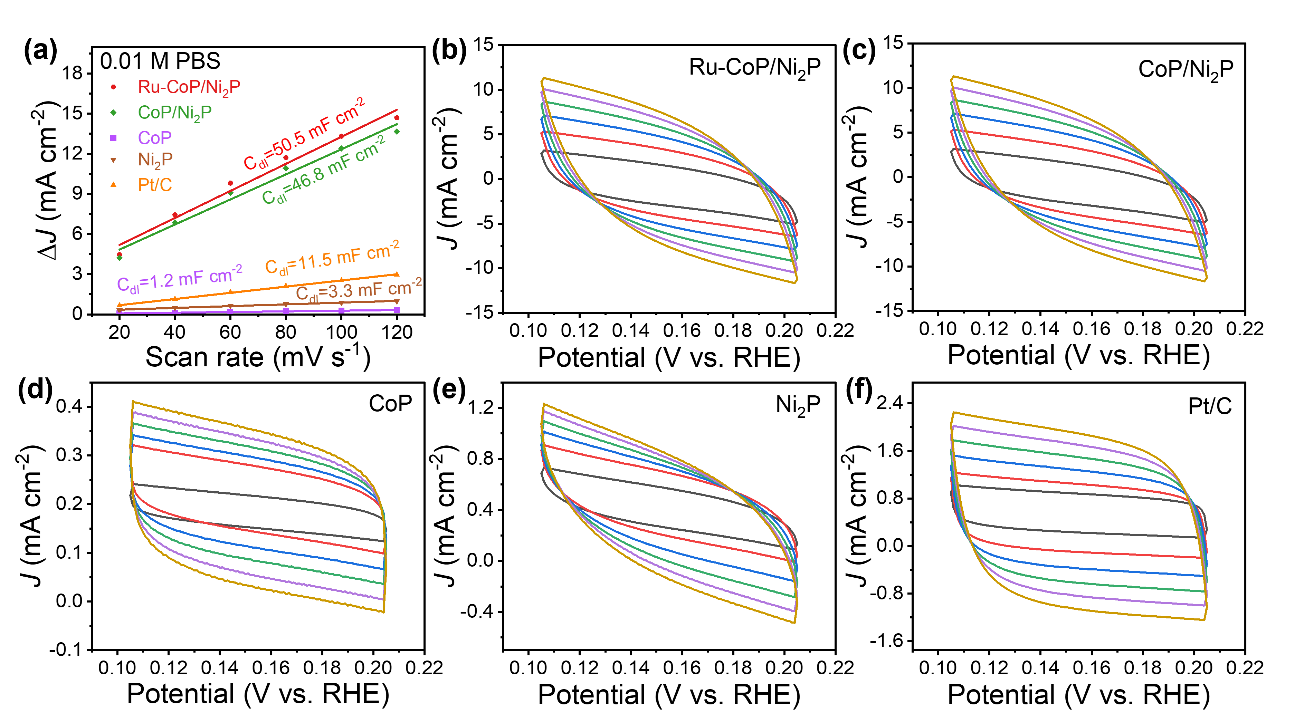


Figure S18. (a) C_dl_ curves of the samples for HER in 0.01 M PBS electrolyte. CVs for (b) Ru-CoP/Ni_2_P, (c) CoP/Ni_2_P, (d) CoP, (e) Ni_2_P, (f) Pt/C, respectively.


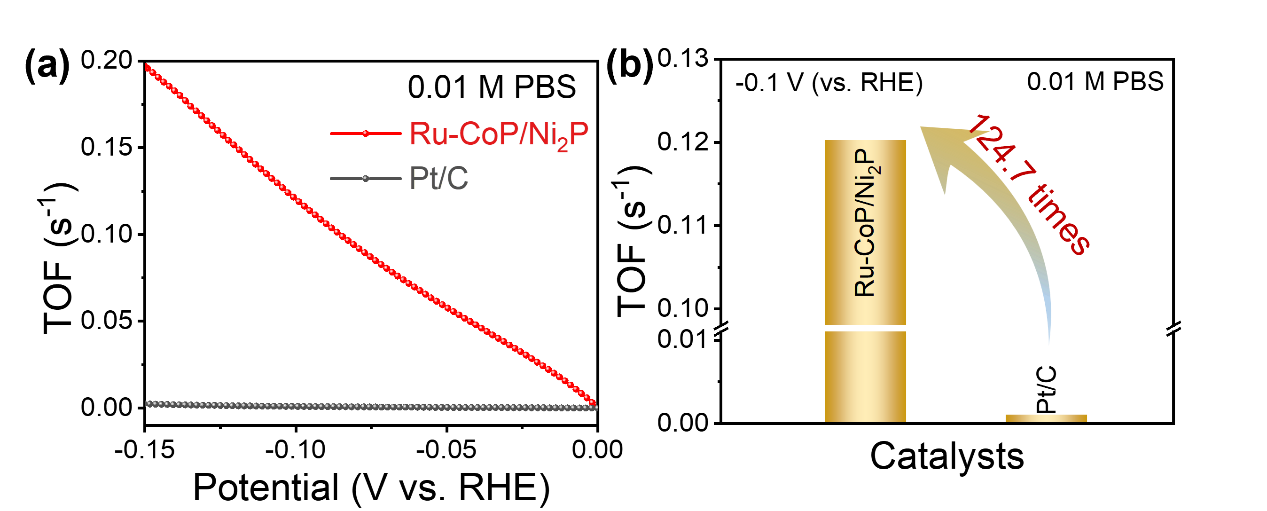


Figure S19. Calculated (a) TOF plots and (b) TOF data at -0.1 V (vs. RHE) of Ru-CoP/Ni_2_P and Pt/C in neutral condition.


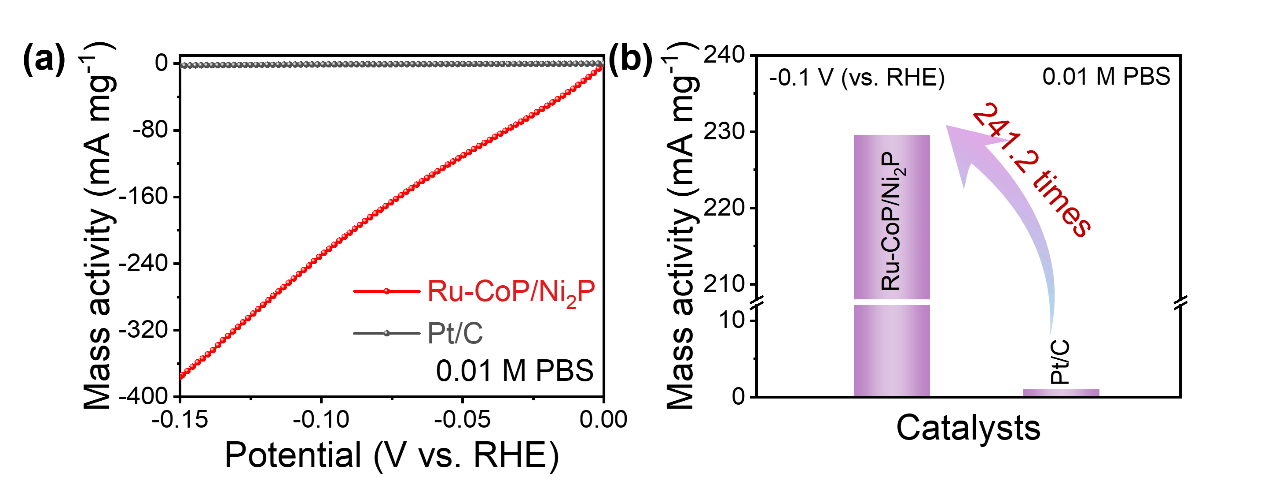


Figure S20. Calculated (a) mass activity plots and (b) mass activity data at -0.1 V (vs. RHE) of of Ru-CoP/Ni_2_P and Pt/C in neutral condition.


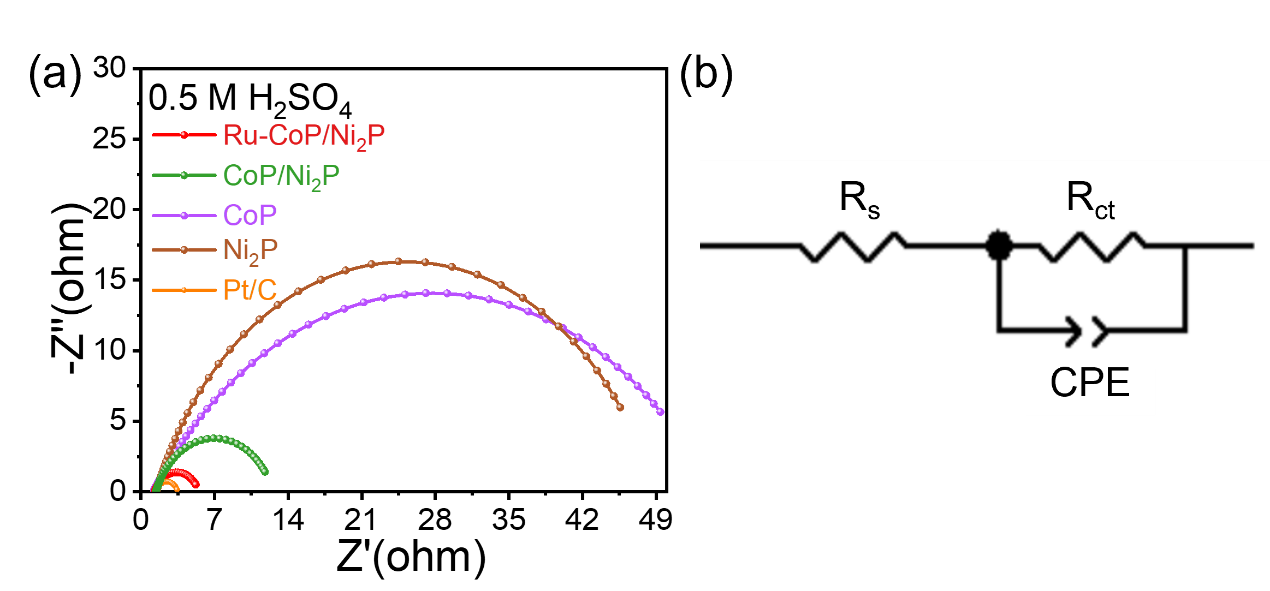


Figure S21. (a) EIS spectra and (b) the equivalent circuit diagram of the samples toward HER in acidic condition.


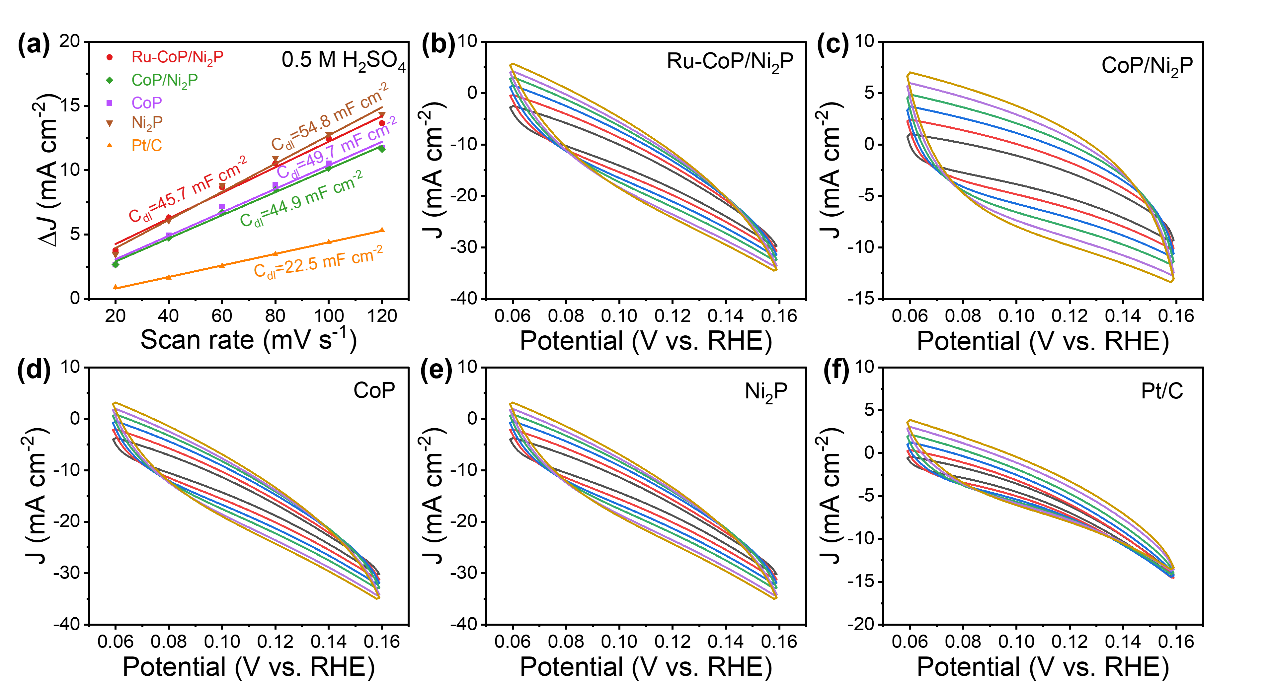


Figure S22. (a) C_dl_ curves of the samples for HER in 0.5 M H_2_SO_4_ electrolyte. CVs for (b) Ru-CoP/Ni_2_P, (c) CoP/Ni_2_P, (d) CoP, (e) Ni_2_P, (f) Pt/C, respectively.


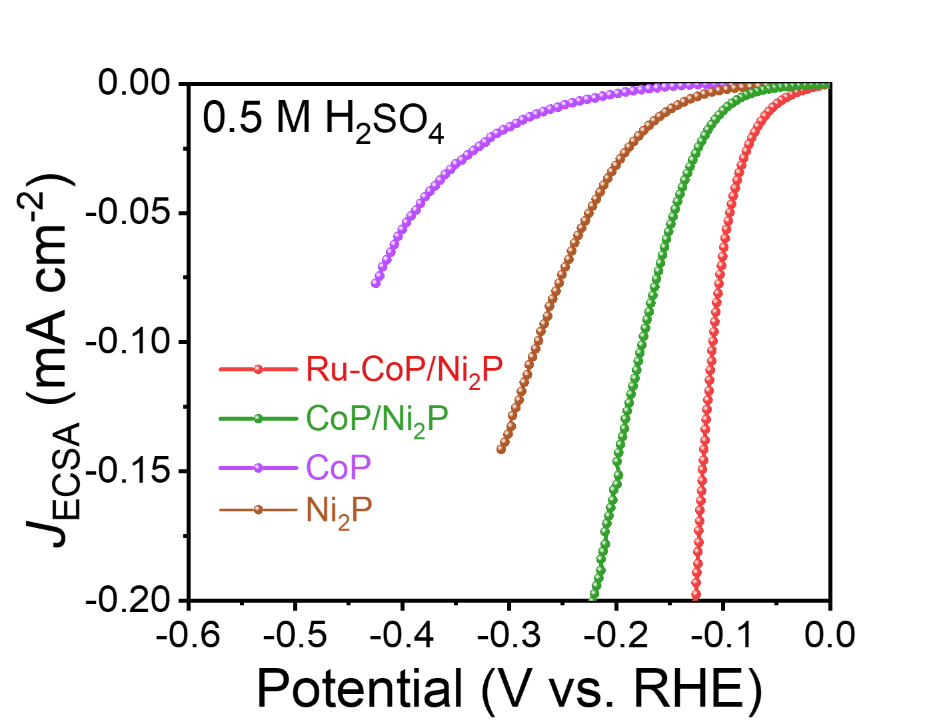


Figure S23. Current density normalized by ECSA of the samples toward HER in acidic condition.


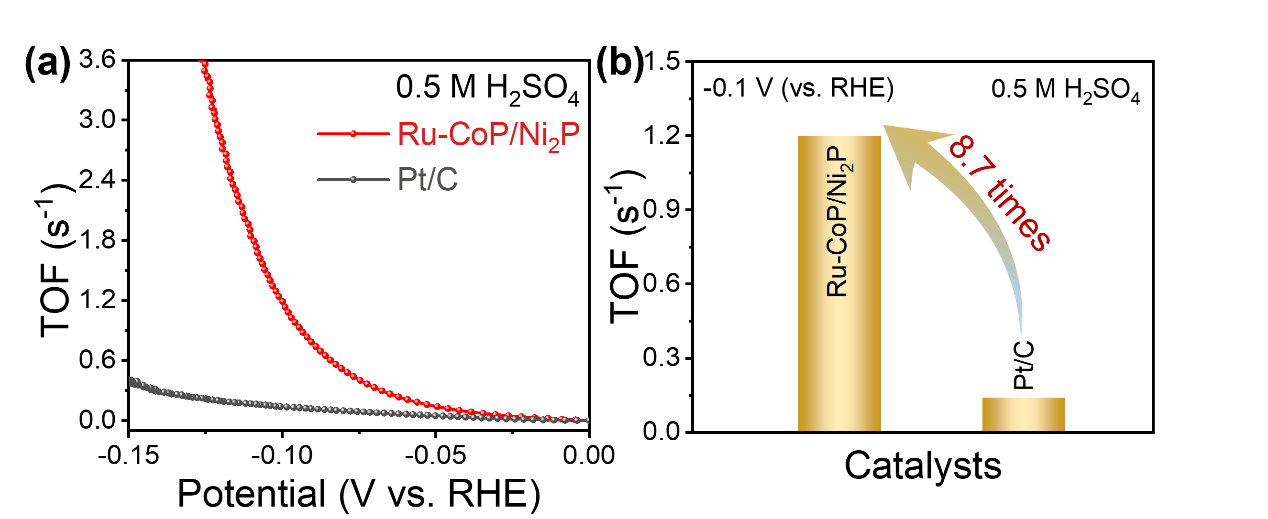


Figure S24. Calculated (a) TOF plots and (b) TOF data at -0.1 V (vs. RHE) of Ru-CoP/Ni_2_P and Pt/C in acidic condition.


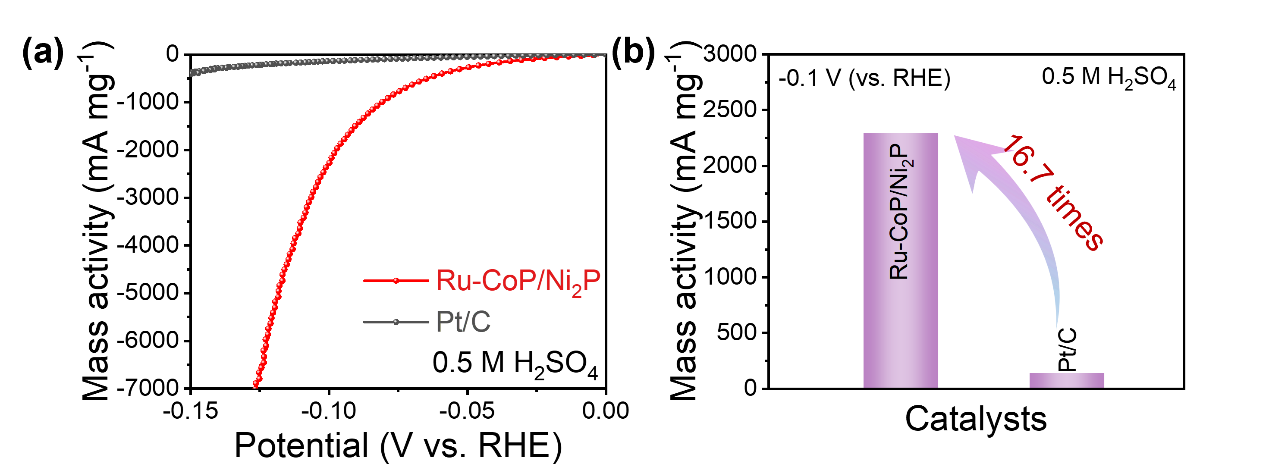


Figure S25. Calculated (a) mass activity plots and (b) mass activity data at -0.1 V (vs. RHE) of of Ru-CoP/Ni_2_P and Pt/C in acidic condition.


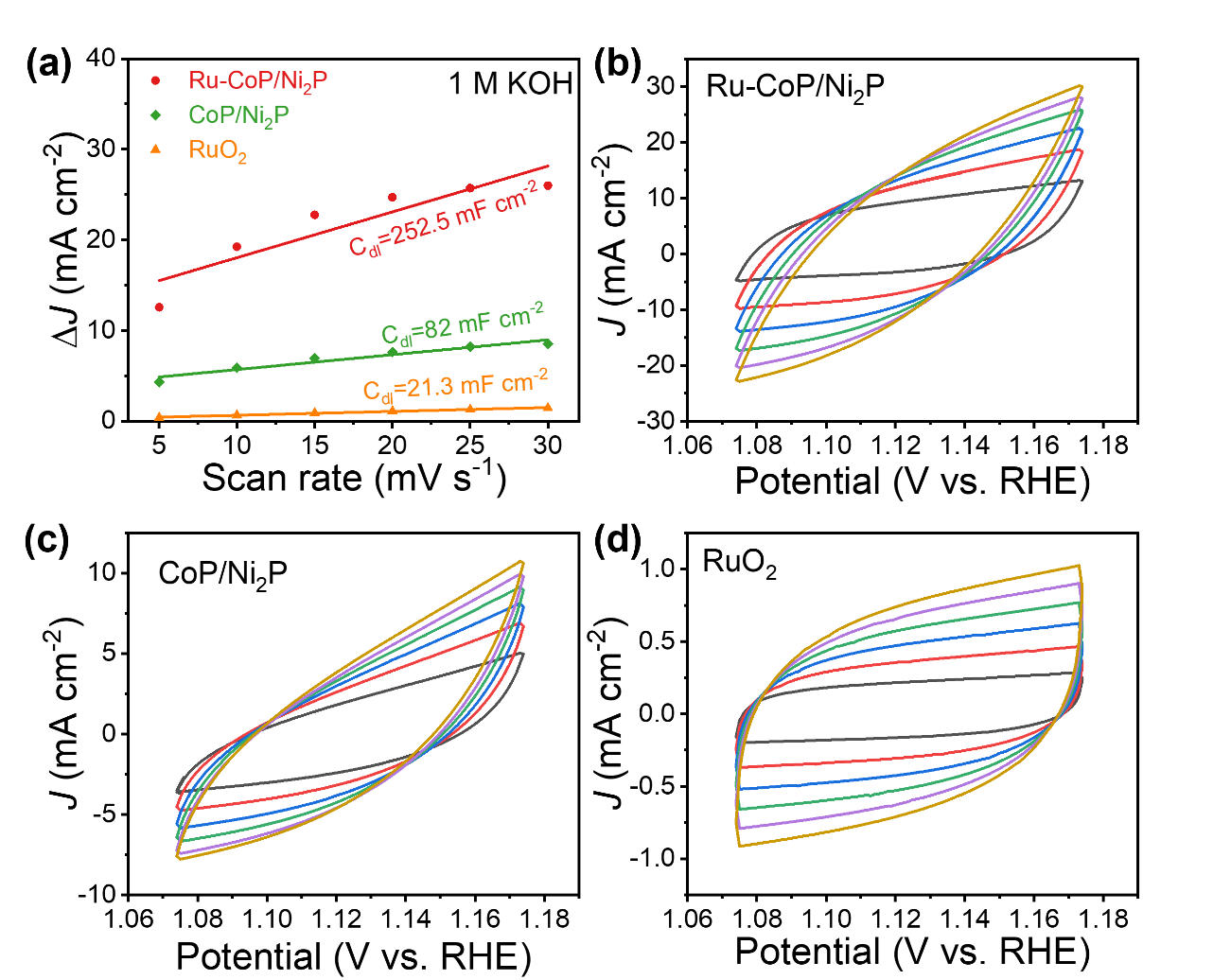


Figure S26. (a) C_dl_ curves of the samples for OER in 1.0 M KOH electrolyte. CVs for (b) Ru-CoP/Ni_2_P, (c) CoP/Ni_2_P, and (d) RuO_2_, respectively.


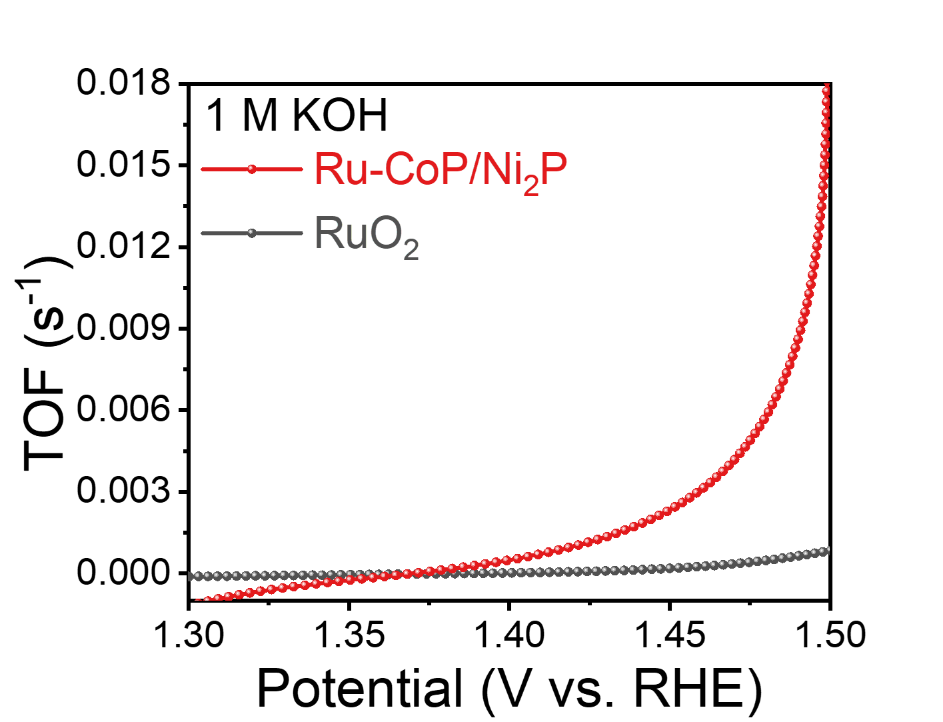


Figure S27. Calculated TOF plots of Ru-CoP/Ni_2_P and RuO_2_ toward OER in neutral condition.


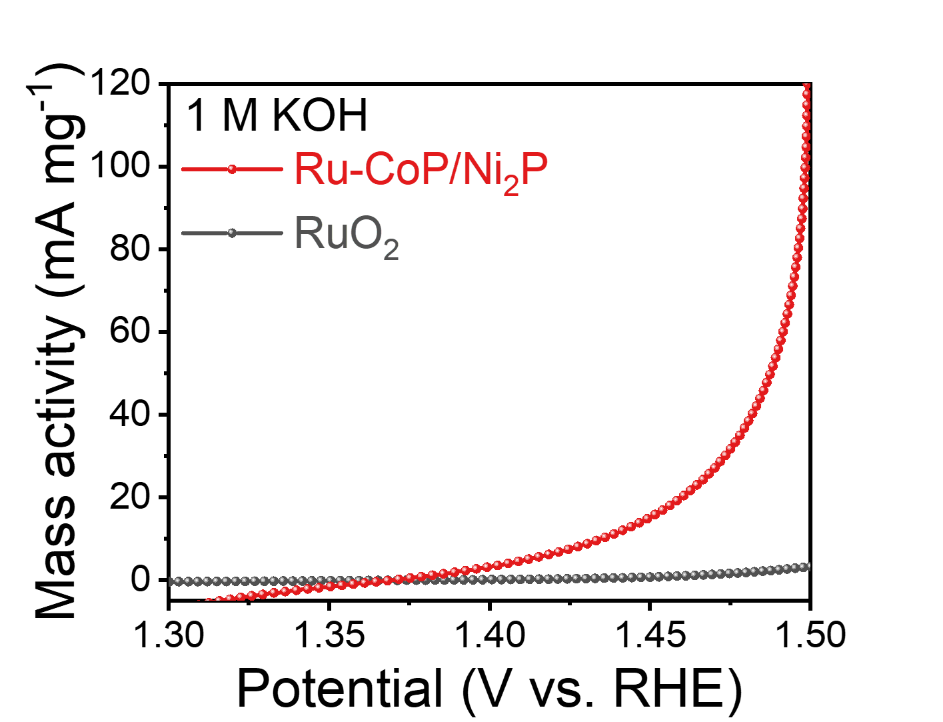


Figure S28. Mass activity plots of Ru-CoP/Ni_2_P and RuO_2_ toward OER in neutral condition.


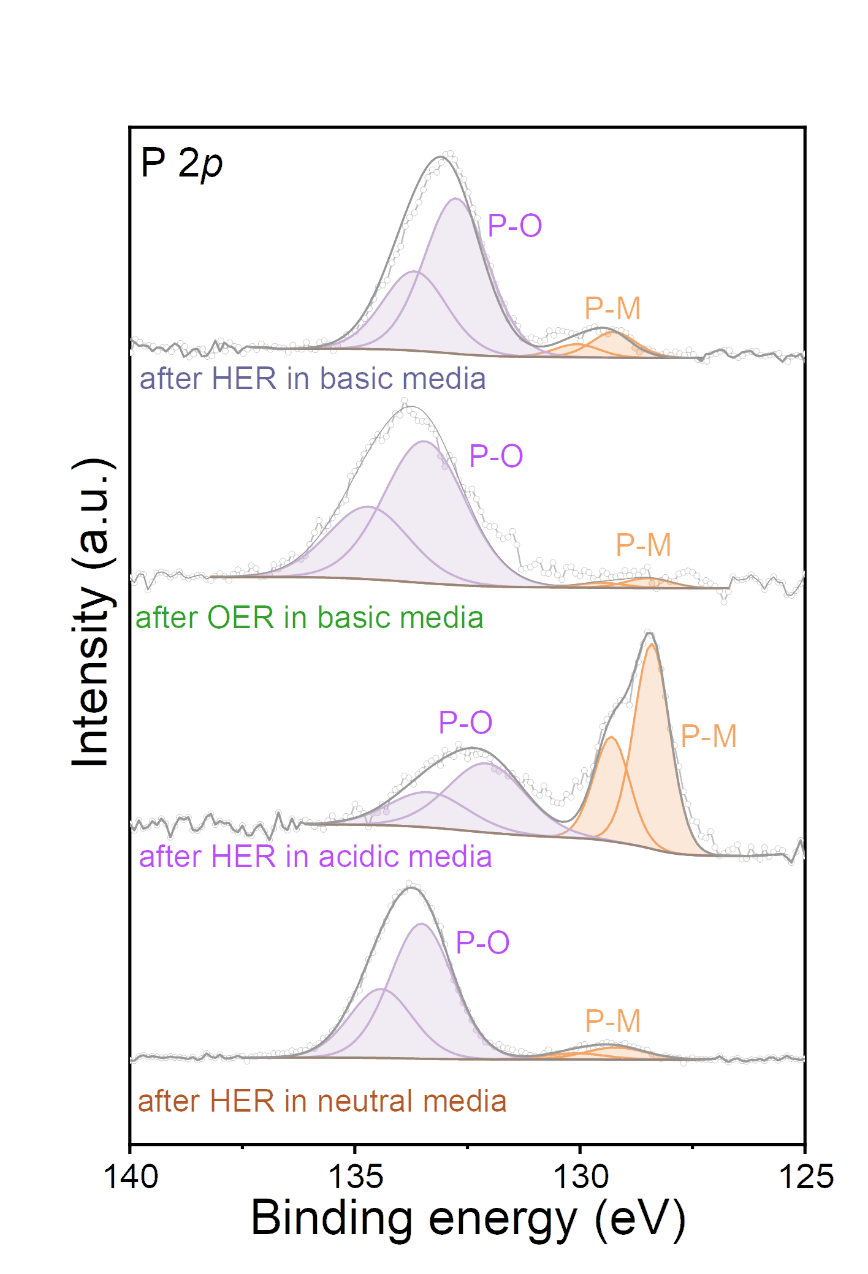


Figure S29. High-resolution P 2*p* spectra of the samples after HER and OER tests.


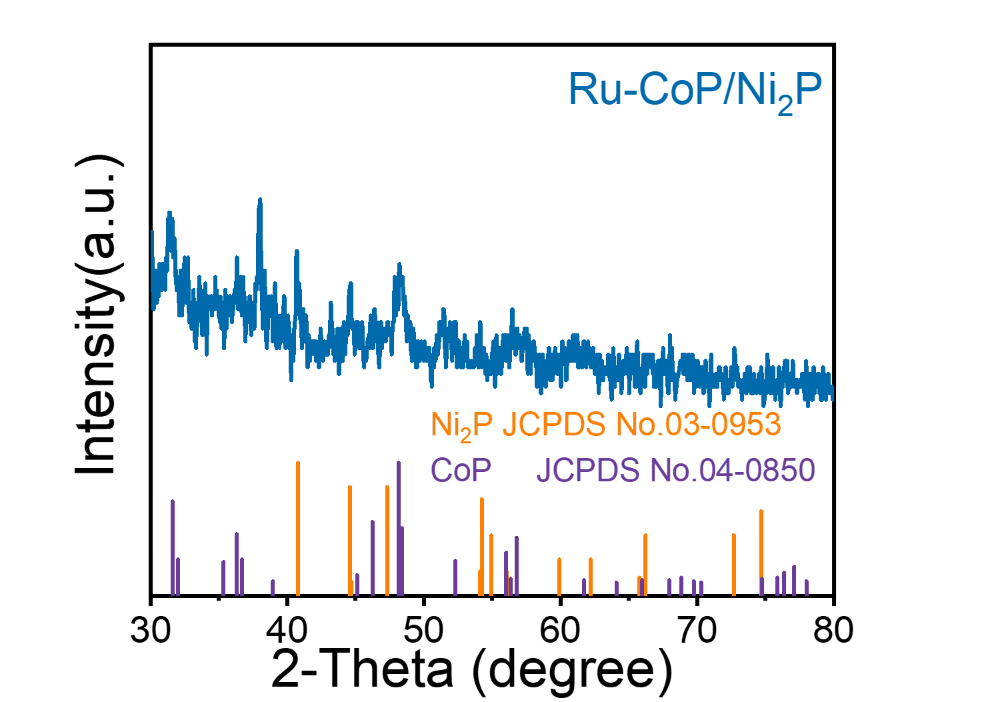


Figure S30. XRD pattern of Ru-CoP/Ni_2_P after OER test.


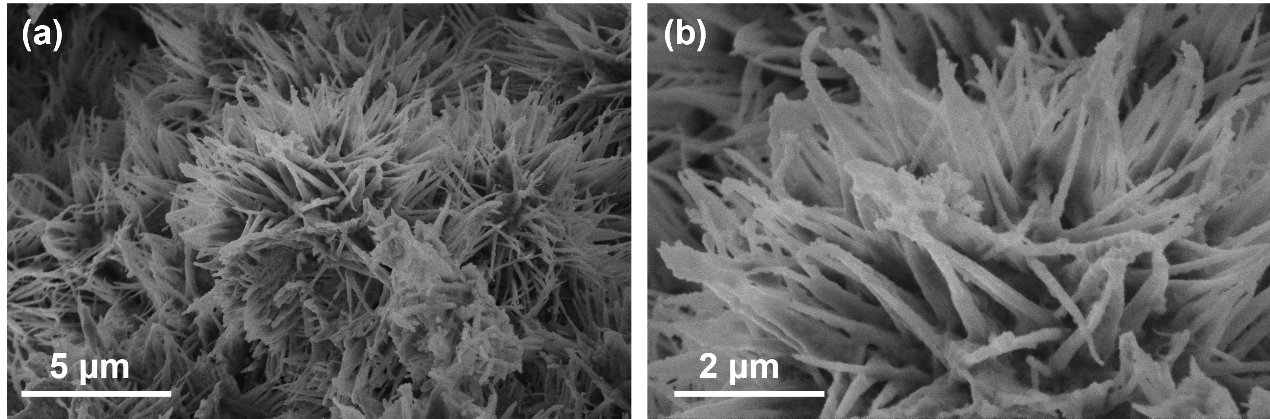


Figure S31. SEM images of Ru-CoP/Ni_2_P after OER with scale bar of (a) 5 μm and (b) 2 μm.


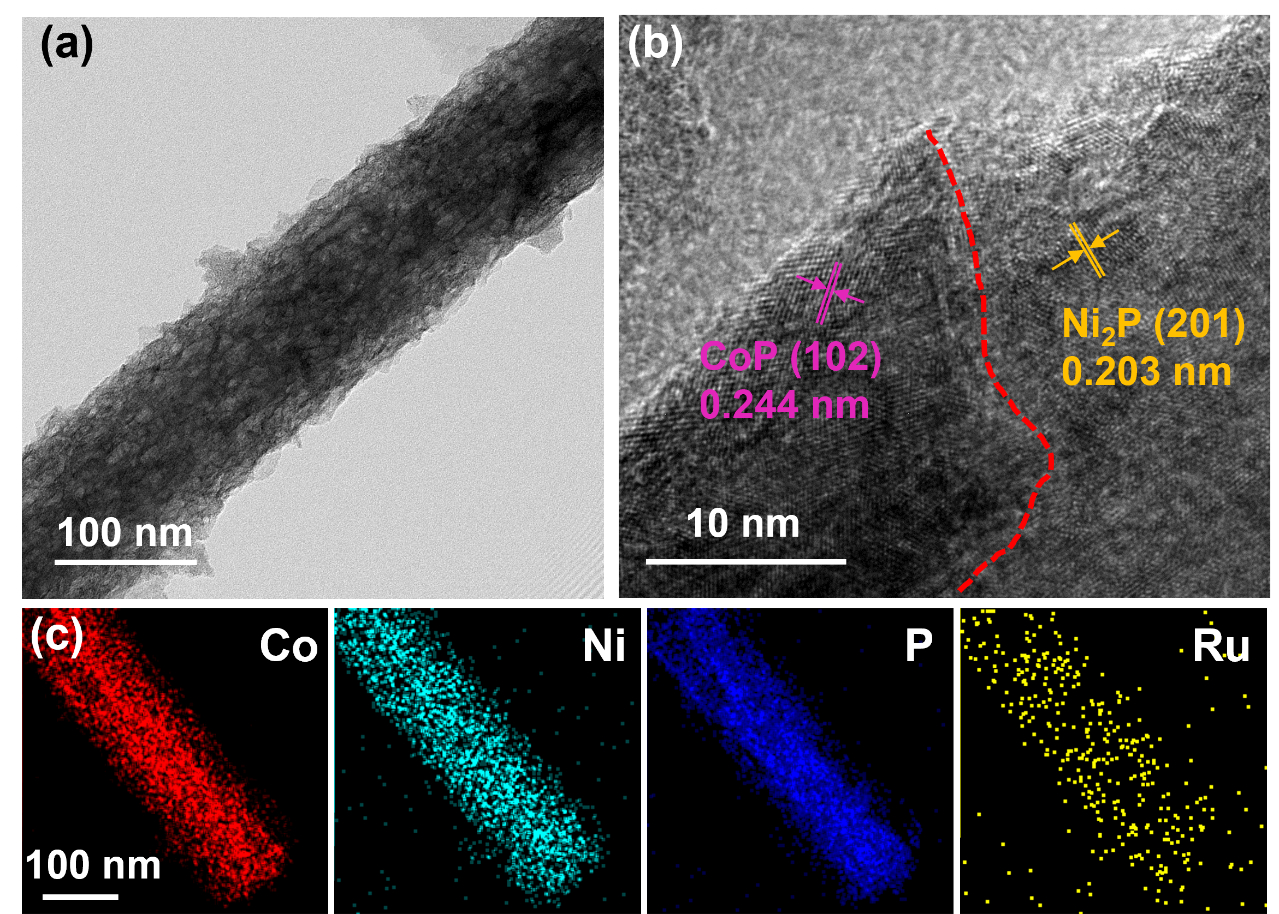


Figure S32. (a) TEM and (b) HRTEM images of Ru-CoP/Ni_2_P after OER test. (c) EDS elemental mapping images of Ru-CoP/Ni_2_P after OER test.


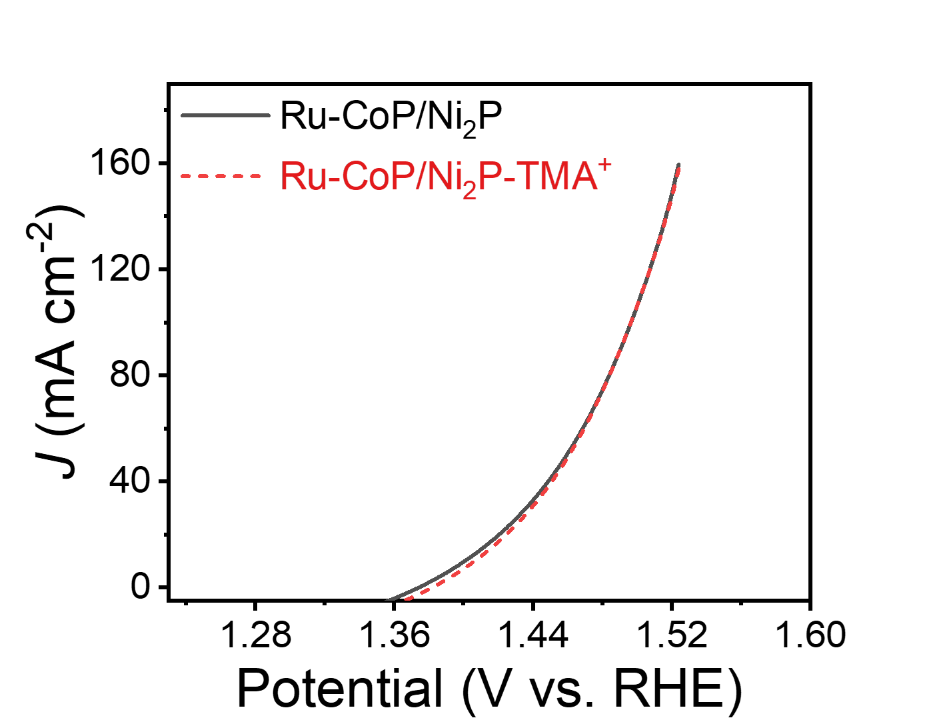


Figure S33. OER LSV curves at different solutions.


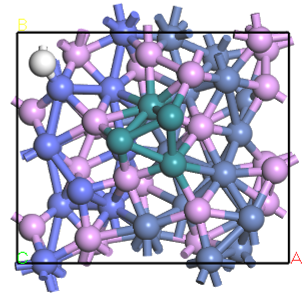


Figure S34. The schematic diagram of absorbed H atoms at Co site.


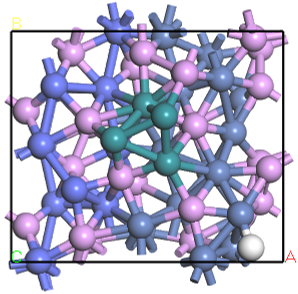


Figure S35. The schematic diagram of absorbed H atoms at Ni site.


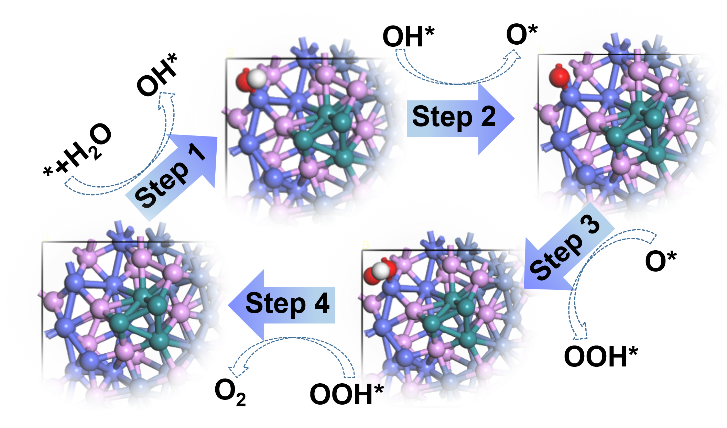


Figure S36. The schematic diagram of alkaline OER elemental steps at Co site.


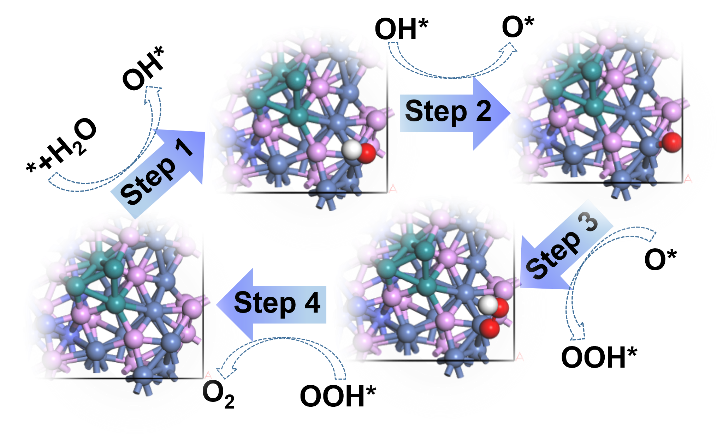


Figure S37. The schematic diagram of alkaline OER elemental steps at Ni site.

**Table S1.** The mass fraction of Ru-CoP/Ni_2_P characterized by ICP-OES.

| **Elements** | **Mass fraction (wt. %)** | **Mass loading (μg cm^-2^)** |
| --- | --- | --- |
| Ru | 2.9 | 33.1 |
| Co | 0.08 | 1250.8 |

**Table S2.** EXAFS fitting parameters at theRu K-edge for various samples.

| **Sample** | **Shell** | **N ^a^** | **R (Å) ^b^** | **σ^2^ (Å^2^·10^-3^) ^c^** | **ΔE_0_ (eV) ^d^** | ***R* factor (%)** |
| --- | --- | --- | --- | --- | --- | --- |
| Ru-CoP/Ni_2_P | Ru-P | 3.4 | 2.32 | 7.9 | 0.1 | 0.9 |
|  | Ru-Ru | 2.8 | 2.66 | 11.1 | -2.4 |  |

*^a^* *N*: coordination numbers; *^b^* *R*: bond distance; *^c^* *σ*^2^: Debye-Waller factors; *^d^* Δ*E*_0_: the inner potential correction. *R* factor: goodness of fit. *Ѕ*02 was set as 0.84/0.86 for Ru-P/Ru-Ru, which was obtained from the experimental EXAFS fit of reference Ru foil/RuO_2_ by fixing CN as the known crystallographic value and was fixed to all the samples.

**Table S3.** The charge transfer resistance obtained from Nyquist plots in 1 M KOH for HER.

| **Samples** | **R_ct_ (Ω)** |
| --- | --- |
| Ru-CoP/Ni_2_P | 3.6 |
| Pt/C | 7.0 |
| CoP/Ni_2_P | 65.0 |
| CoP | 67.0 |
| Ni_2_P | 252.2 |

**Table S4.** The calculated ECSA values of different samples for HER in 1.0 M KOH.

| **Samples** | **ECSA** |
| --- | --- |
| Ru-CoP/Ni_2_P | 1147.5 |
| CoP/Ni_2_P | 762.5 |
| CoP | 420 |
| Ni_2_P | 85 |
| Pt/C | 315 |

**Table S5.** Comparisons overpotential (η_10_) between Ru-CoP/Ni_2_P and the reported HER electrocatalysts in alkaline condition.

| **Catalysts** | **Overpotential (mV) at 10 mA cm^-2^** | **Reference** |
| --- | --- | --- |
| Ru-CoP/Ni_2_P | 64 | This work |
| CoMoP@C | 81 | [3] |
| CoP_x_/N-rGO | 104 | [4] |
| CoNiS_x_/NF-25 | 123 | [5] |
| Ru/C_3_N_4_/C | 79 | [6] |
| [hcp-Ru@NC-700](mailto:hcp-Ru@NC-700) | 120 | [7] |
| [CoP@CoOOH/CP](mailto:CoP@CoOOH/CP) | 87.5 | [8] |
| [Cu_2-x_S@Ru NPs](mailto:Cu2-xS@Ru%20NPs) | 82 | [9] |
| SA-Ru-MoS_2_ | 76 | [10] |

**Table S6.** The charge transfer resistance obtained from Nyquist plots in 0.01 M PBS for HER.

| **Samples** | **R_ct_ (Ω)** |
| --- | --- |
| Ru-CoP/Ni_2_P | 7.0 |
| Pt/C | 9.8 |
| CoP/Ni_2_P | 6.9 |
| CoP | 87.0 |
| Ni_2_P | 23.7 |

**Table S7.** The calculated ECSA values of different samples for HER in 0.01 M PBS.

| **Samples** | **ECSA** |
| --- | --- |
| Ru-CoP/Ni_2_P | 1262.5 |
| CoP/Ni_2_P | 1170 |
| CoP | 30 |
| Ni_2_P | 82.5 |
| Pt/C | 287.5 |

**Table S8.** The charge transfer resistance obtained from Nyquist plots in 0.5 M H_2_SO_4_ for HER.

| **Samples** | **R_ct_ (Ω)** |
| --- | --- |
| Ru-CoP/Ni_2_P | 4.2 |
| Pt/C | 2.1 |
| CoP/Ni_2_P | 11.2 |
| CoP | 53.1 |
| Ni_2_P | 47.4 |

**Table S9.** The calculated ECSA values of different samples for HER in 0.5 M H_2_SO_4_.

| **Samples** | **ECSA** |
| --- | --- |
| Ru-CoP/Ni_2_P | 1142.5 |
| CoP/Ni_2_P | 1122.5 |
| CoP | 1242.5 |
| Ni_2_P | 1370 |
| Pt/C | 562.5 |

**Table S10.** Comparisons overpotential (η_10_) between Ru-CoP/Ni_2_P and the reported HER electrocatalysts in neutral condition.

| **Catalysts** | **Overpotential (mV) at 10 mA cm^-2^** | **Reference** |
| --- | --- | --- |
| Ru-CoP/Ni_2_P | 125 | This work |
| RuP_2_@NC | 196 | [11] |
| Ru/C-2 | 188 | [12] |
| RuNi@CN-700 | 144 | [13] |
| IrO_2_-RuO_2_/C | 147 | [14] |
| IrO_2_/V_2_O_5_ | 176 | [15] |
| Co-Ni-B | 133 | [16] |
| Co-Fe-P | 138 | [17] |
| MoP NA/CC | 187 | [18] |
| Mo/Mo_2_C/N-CNFs | 294 | [19] |
| Fe_x_Co_3-x_(PO_4_)_2_/Cu | 291.5 | [20] |

**Table S11.** Comparisons overpotential (η_10_) between Ru-CoP/Ni_2_P and the reported HER electrocatalysts in acidic condition.

| **Catalysts** | **Overpotential (mV) at 10 mA cm^-2^** | **Reference** |
| --- | --- | --- |
| Ru-CoP/Ni_2_P | 53 | This work |
| Ru@Co/N-CNTs-2 | 92 | [21] |
| Cu_2-x_S@Ru NPs | 129 | [9] |
| Co@NC-600 | 279 | [22] |
| C_3_N_4_–Ru–F | 140 | [23] |
| Ru-MoS_2_ | 300 | [24] |
| 1D-RuO_2_-CNx | 93 | [25] |
| IrO_2_/V_2_O_5_ | 65 | [15] |
| IrO_2_-RuO_2_/C | 82 | [14] |

**Table S12.** The charge transfer resistance obtained from Nyquist plots in 1 M KOH for OER.

| **Samples** | **R_ct_ (****Ω)** |
| --- | --- |
| Ru-CoP/Ni_2_P | 0.5 |
| RuO_2_ | 7.4 |
| CoP/Ni_2_P | 1.6 |

**Table S13.** The calculated ECSA values of different samples for OER in 1.0 M KOH.

| **Samples** | **ECSA** |
| --- | --- |
| Ru-CoP/Ni_2_P | 6312.5 |
| CoP/Ni_2_P | 2050 |
| RuO_2_ | 532.5 |

**Table S14.** Comparisons overpotential (η_10_) between Ru-CoP/Ni_2_P and the reported OER electrocatalysts in acidic condition.

| **Catalysts** | **Overpotential (mV) at 10 mA cm^-2^** | **Reference** |
| --- | --- | --- |
| Ru-CoP/Ni_2_P | 196 | This work |
| [Ir@Ni-NDC](mailto:Ir@Ni-NDC) | 210 | [26] |
| IrO_2_-RuO_2_/C | 270 | [14] |
| Ir-NSs | 266 | [27] |
| a/c RuO_2_ | 287 | [28] |
| RuSAs/SC-FeCoNi | 205 | [29] |
| Au@Ni_2_P-350 | 240 | [30] |
| Ir-Ni(OH)_2_ | 260 | [31] |
| RuCu NSs | 234 | [32] |
| AN-CuNiFe | 224 | [33] |

**Table S15.** Comparisons cell voltage of overall water splitting at 10 mA cm^-2^ between Ru-CoP/Ni_2_P and the reported electrocatalysts in 1.0 M KOH condition.

| **Systems** | **Cell voltage (V)** | **Reference** |
| --- | --- | --- |
| Ru-CoP/Ni_2_P \|\| Ru-CoP/Ni_2_P | 1.51 | This work |
| MXene@RuCo NPs \|\| MXene@RuCo NPs | 1.52 | [34] |
| Co_9_S_8_@NOSC-900 \|\| Co_9_S_8_@NOSC-900 | 1.6 | [35] |
| Co-MoS_2_/BCCF-21 \|\| Co-MoS_2_/BCCF-21 | 1.55 | [36] |
| Ru-VO_2_ \|\| Ru-VO_2_ | 1.54 | [37] |
| P-CoMo_2_S_4_/Co_4_S_3_-Co_2_P \|\| P-CoMo_2_S_4_/Co_4_S_3_-Co_2_P | 1.55 | [38] |
| Fe-CoP@CC \|\| Fe-CoP@CC | 1.58 | [39] |
| Co-P-S \|\| Co-P-S | 1.6 | [40] |
| Ru_0.7_Co_0.3_ aerogel/CC//Ru aerogel/CC | 1.587 | [41] |
| CoP@CoOOH/CP | 1.52 | [8] |

**References**

[1] H. Huang, L. Fu, W. Kong, H. Ma, X. Zhang, J. Cai, S. Wang, Z. Xie, S. Xie, *Small* **2022**, 18, 2201333.

[2] J. Kim, H. J. Kim, B. Ruqia, M. J. Kim, Y. J. Jang, T. H. Jo, H. Baik, H. S. Oh, H. S. Chung, K. Baek, S. Noh, M. Jung, K. J. Kim, H. K. Lim, Y. S. Youn, S. I. Choi, *Adv. Mater.* **2021**, 33, 2105248.

[3] Y.-Y. Ma, C.-X. Wu, X.-J. Feng, H.-Q. Tan, L.-K. Yan, Y. Liu, Z.-H. Kang, E.-B. Wang, Y.-G. Li, *Energy Environ. Sci.* **2017**, 10, 788.

[4] L. Zheng, W. Hu, X. Shu, H. Zheng, X. Fang, *Adv. Mater. Interfaces* **2018**, 5, 1800515.

[5] W. Lu, X. Li, F. Wei, K. Cheng, W. Li, Y. Zhou, W. Zheng, L. Pan, G. Zhang, *ACS Sustain. Chem. Eng.* **2019**, 7, 12501.

[6] Y. Zheng, Y. Jiao, Y. Zhu, L. H. Li, Y. Han, Y. Chen, M. Jaroniec, S.-Z. Qiao, *J. Am. Chem. Soc.* **2016**, 138, 16174.

[7] Y. Li, L. A. Zhang, Y. Qin, F. Chu, Y. Kong, Y. Tao, Y. Li, Y. Bu, D. Ding, M. Liu, *ACS Catal.* **2018**, 8, 5714.

[8] B. Zhang, J. Shan, W. Wang, P. Tsiakaras, Y. Li, *Small* **2022**, 18, 2106012.

[9] D. Yoon, J. Lee, B. Seo, B. Kim, H. Baik, S. H. Joo, K. Lee, *Small* **2017**, 13, 1700052.

[10] J. Zhang, X. Xu, L. Yang, D. Cheng, D. Cao, *Small Methods* **2019**, 3, 1900653.

[11] B.-Y. Guo, X.-Y. Zhang, J.-Y. Xie, Y.-H. Shan, R.-Y. Fan, W.-L. Yu, M.-X. Li, D.-P. Liu, Y.-M. Chai, B. Dong, *Int. J. Hydrogen Energy* **2021**, 46, 7964.

[12] H. Shi, L. Liu, Y. Shi, F. Liao, Y. Li, M. Shao, *Int. J. Hydrogen Energy* **2019**, 44, 11817.

[13] W. Wang, J. Peng, L. Yang, Q. Liu, Y. Wang, H. Liu, *Int. J. Electrochem. Sci.* **2020**, 15, 11769.

[14] R. Samanta, P. Panda, R. Mishra, S. Barman, *Energ. Fuel.* **2022**, 36, 1015.

[15] X. Zheng, M. Qin, S. Ma, Y. Chen, H. Ning, R. Yang, S. Mao, Y. Wang, *Adv. Sci.* **2022**, 9, 2104636.

[16] M. D. Sharma, C. Mahala, M. Basu, *Inorg. Chem.* **2020**, 59, 4377.

[17] J. Chen, J. Liu, J.-Q. Xie, H. Ye, X.-Z. Fu, R. Sun, C.-P. Wong, *Nano Energy* **2019**, 56, 225.

[18] Z. Pu, S. Wei, Z. Chen, S. Mu, *Appl. Catal. B Environ. Energy* **2016**, 196, 193.

[19] M. Li, H. Wang, Y. Zhu, D. Tian, C. Wang, X. Lu, *Appl. Surf. Sci.* **2019**, 496, 143672.

[20] C. Yang, T. He, W. Zhou, R. Deng, Q. Zhang, *ACS Sustainable Chemistry & Engineering* **2020**, 8, 13793.

[21] Z. Liu, X. Yang, G. Hu, L. Feng, *ACS Sustainable Chem. Eng.* **2020**, 8, 9136.

[22] P. Jiang, J. Chen, C. Wang, K. Yang, S. Gong, S. Liu, Z. Lin, M. Li, G. Xia, Y. Yang, J. Su, Q. Chen, *Adv. Mater.* **2018**, 30, 1705324.

[23] Y. Peng, B. Lu, L. Chen, N. Wang, J. E. Lu, Y. Ping, S. Chen, *J. Mater. Chem. A* **2017**, 5, 18261.

[24] Y. Cheng, S. Lu, F. Liao, L. Liu, Y. Li, M. Shao, *Adv. Funct. Mater.* **2017**, 27, 1700359.

[25] T. Bhowmik, M. K. Kundu, S. Barman, *ACS Appl. Mater. Interfaces* **2016**, 8, 28678.

[26] J. Yang, Y. Shen, Y. Sun, J. Xian, Y. Long, G. Li, *Angew. Chem. Int. Ed.* **2023**, 62, e202302220.

[27] Z. Cheng, B. Huang, Y. Pi, L. Li, Q. Shao, X. Huang, *Natl. Sci. Rev.* **2020**, 7, 1340.

[28] L. Zhang, H. Jang, H. Liu, M. G. Kim, D. Yang, S. Liu, X. Liu, J. Cho, *Angew. Chem. Int. Ed.* **2021**, 60, 18821.

[29] Y. Hu, G. Luo, L. Wang, X. Liu, Y. Qu, Y. Zhou, F. Zhou, Z. Li, Y. Li, T. Yao, C. Xiong, B. Yang, Z. Yu, Y. Wu, *Adv. Energy Mater.* **2021**, 11, 2002816.

[30] C. Cai, S. Han, Q. Wang, M. Gu, *ACS Nano* **2019**, 13, 8865.

[31] Q. He, S. Qiao, Q. Zhou, Y. Zhou, H. Shou, P. Zhang, W. Xu, D. Liu, S. Chen, X. Wu, L. Song, *Nano Lett.* **2022**, 22, 3832.

[32] Q. Yao, B. Huang, N. Zhang, M. Sun, Q. Shao, X. Huang, *Angew. Chem. Int. Ed.* **2019**, 58, 13983.

[33] Z. Cai, L. Li, Y. Zhang, Z. Yang, J. Yang, Y. Guo, L. Guo, *Angew. Chem. Int. Ed.* **2019**, 131, 4233.

[34] J. Li, C. Hou, C. Chen, W. Ma, Q. Li, L. Hu, X. Lv, J. Dang, *ACS Nano* **2023**, 17, 10947.

[35] S. Huang, Y. Meng, S. He, A. Goswami, Q. Wu, J. Li, S. Tong, T. Asefa, M. Wu, *Adv. Funct. Mater.* **2017**, 27, 1606585.

[36] Q. Xiong, Y. Wang, P. F. Liu, L. R. Zheng, G. Wang, H. G. Yang, P. K. Wong, H. Zhang, H. Zhao, *Adv. Mater.* **2018**, 30, 1801450.

[37] Z. Niu, Z. Lu, Z. Qiao, S. Wang, X. Cao, X. Chen, J. Yun, L. Zheng, D. Cao, *Adv. Mater.* **2023**, 36, 2310690.

[38] K. Dong, D. T. Tran, X. Li, S. Prabhakaran, D. H. Kim, N. H. Kim, J. H. Lee, *Appl. Catal. B: Environ. Energy* **2024**, 344, 123649.

[39] Y. Yang, H. Meng, C. Kong, W. Ma, H. Zhu, F. Ma, C. Wang, Z. Hu, *Int. J. Hydrogen Energy* **2021**, 46, 28053.

[40] F. Du, Y. Zhang, H. He, T. Li, G. Wen, Y. Zhou, Z. Zou, *J. Power Sources* **2019**, 431, 182.

[41] Z. Lin, S. Liu, Y. Liu, Z. Liu, S. Zhang, X. Zhang, Y. Tian, Z. Tang, *J. Power Sources* **2021**, 514, 230600.
